# Zebrafish as a Model to Study the Role of Peroxisome Proliferating-Activated Receptors in Adipogenesis and Obesity

**DOI:** 10.1155/2015/358029

**Published:** 2015-11-30

**Authors:** Marjo J. Den Broeder, Victoria A. Kopylova, Leonie M. Kamminga, Juliette Legler

**Affiliations:** ^1^Institute for Environmental Studies, Faculty of Earth and Life Sciences, VU University, 1081 HV Amsterdam, Netherlands; ^2^Department of Molecular Biology, Faculty of Sciences, Radboud Institute for Molecular Life Science, Radboud University, Nijmegen, Netherlands; ^3^Radboud University Nijmegen Medical Center, Nijmegen, Netherlands

## Abstract

The Peroxisome Proliferator-Activated Receptors (PPARs) PPARA and PPARD are regulators of lipid metabolism with important roles in energy release through lipid breakdown, while PPARG plays a key role in lipid storage and adipogenesis. The aim of this review is to describe the role of PPARs in lipid metabolism, adipogenesis, and obesity and evaluate the zebrafish as an emerging vertebrate model to study the function of PPARs. Zebrafish are an appropriate model to study human diseases, including obesity and related metabolic diseases, as pathways important for adipogenesis and lipid metabolism which are conserved between mammals and fish. This review synthesizes knowledge on the role of PPARs in zebrafish and focuses on the putative function of PPARs in zebrafish adipogenesis. Using* in silico* analysis, we confirm the presence of five PPARs (*pparaa*,* pparab*,* pparda*,* ppardb*, and* pparg*) in the zebrafish genome with 67–74% identity to human and mouse PPARs. During development,* pparda/b* paralogs and* pparg* show mRNA expression around the swim bladder and pancreas, the region where adipocytes first develop, whereas* pparg* is detectable in adipocytes at 15 days post fertilization (dpf). This review indicates that the zebrafish is a promising model to investigate the specific functions of PPARs in adipogenesis and obesity.

## 1. Introduction

In the last 30 years, obesity has become a worldwide epidemic, and according to the World Health Organization (WHO), over 700 million people can be characterized as obese [1]. Obesity is a risk factor for developing type II diabetes mellitus, cardiovascular diseases and hypertension, as well as cancer. Over the last decade, many studies have described the important biological functions of Peroxisome Proliferator-Activated Receptors (PPARs) and their role in obesity. Genetic variation in PPARs results in altered fat deposition and body weight, as studies on the development of obesity, T2DM, dyslipidemia, and cardiovascular diseases have identified single nucleotide polymorphisms (SNPs) in PPAR genes [2]. PPARs are important factors for adipocyte differentiation and energy homeostasis and are highly expressed in tissue with active lipid metabolism. In addition, PPARs are involved in embryonic development, cell differentiation, and inflammation [3–5].

The aim of this review is to describe the function of PPARs in lipid metabolism and adipogenesis, in particular their role in obesity and related disorders. As numerous animal models have been used to study the origin of obesity and to gain better knowledge of PPAR-related molecular mechanisms, we evaluate the zebrafish as an emerging model to study adipogenesis. We hypothesize that PPARs in zebrafish have similar functions as in mammals.

*(1) Peroxisome Proliferator-Activated Receptors.* PPARs are nuclear hormone receptors which belong to the NR1C subfamily of steroid receptor superfamily. The classifications of PPARs in the nuclear receptor family are described in a nomenclature system according to Nuclear Receptors Nomenclature Committee [6]. Three PPARs have been identified in vertebrates, namely, PPARA (hPPAR, PPAR*α*, and NR1C1), PPARD (NR1C2, PPAR*β*/*δ*, FAAR, NUCI, and NUCII), and PPARG (NR1C3, PPAR*γ*) which are encoded by different genes [4]. The zebrafish orthologs of PPARA, PPARD, and PPARG are referred to as Pparaa, Pparab, Pparda, Ppardb, and Pparg according to the ZFIN nomenclature (http://zfin.org/).

Like other nuclear receptors, PPARs have a protein structure that generally consists of four parts, namely, an N-terminal domain (NTD), a DNA-binding domain (DBD), a ligand binding domain (LBD), and a connective structure (hinge) [7, 8]. The NTD contains a ligand-independent activation factor-1 (AF-1). The DBD consists of two zinc fingers that specifically bind to the peroxisome proliferator response element (PPRE) in the promotor regions of PPAR target genes [9]. The C-terminal part of the protein, the LBD, consists of 13 *α*-helices and 4 *β*-sheets which form the ligand binding pocket. The LBD contains the ligand dependent activation factor-2 (AF-2), and ligand binding to AF-2 results in activation of the LBD [3, 10]. Expression of PPARs is predominantly in the nucleus, though PPARA and PPARG are also found in lower concentrations in the cytoplasm as well [11, 12]. PPARs can be shuttled between the cytoplasm and the nucleus by export receptors that recognize two different nuclear localization signals (NLSs) on the PPAR protein [12].

PPARs regulate transcriptional gene activation through heterodimerization with retinoid X receptors (RXR) [13, 14]. All PPARs can form a complex with RXR, and heterodimerization between PPAR and RXR is ligand independent [15, 16]. In the absence of ligands, PPAR:RXR heterodimers bound to PPREs will act as a transcriptional repressor due to binding of corepressor proteins such as nuclear receptor corepressor 1 (NCoR) and silencing mediator of retinoic acid and thyroid hormone receptor (SMRT). Binding of a specific PPAR agonist to LBD leads to the release of corepressor complex and recruitment of the coactivation factors. Coactivators of PPARs include the steroid receptor coactivator-1 (SRC-1), CREB-binding protein (CBP), PPAR-binding protein (PBP), P300, cyclin G2, PPAR-interacting protein (PRIP), and PPAR*γ* coactivator-1 (PGC-1) [17–19]. As a consequence of a conformational change of the LBD, the PPAR:RXR heterodimer binds to the PPRE present in promoter regions in order to regulate transcription of target genes by facilitating RNA polymerase II function. The PPRE consists of direct repeats (DRs) containing two hexanucleotide sequences AGGTCA which are separated by one nucleotide [20]. The 5′ flanking site of the PPRE is important for PPAR isoform:RXR heterodimer binding specificity and consists of a 7-nucleotide sequence consensus (C[A/G][A/G]A[A/T]CT) [21].

The LBD of the PPARs is activated by specific endogenous agonists such as fatty acids, fatty acids derivatives, phospholipids, eicosanoids, and prostaglandins (Table 1). Many synthetic ligands of PPARS have been developed for the treatment of diabetes mellitus and high levels of triglycerides and cholesterol, such as the thiazolidine drugs (TZDs) and fibrates (Table 1). Recently, chemicals present in the environment through human activity such as pesticides and phthalate ester plasticizers have also been identified as PPAR ligands [22, 23].

*(2) Biological Function of PPARs in Lipid Metabolism and Adipocyte Differentiation.* PPARA is mainly involved in fatty acid oxidation and is highly expressed in tissue with mitochondrial and peroxisomal *β*-oxidation such as brown adipose tissue (BAT), liver, and to a lower extent also in heart, kidney, and muscles [24]. PPARA is activated by long chain unsaturated fatty acids, eicosanoids, and synthetic fibrates, which have been developed to treat dyslipidemia by reducing triglyceride levels [25, 26].

PPARD is ubiquitously expressed and has a common function in fatty acid metabolism comparable to PPARA. PPARD also seems to be involved in embryo implantation, keratinocyte differentiation, and wound healing [27, 28]. PPARD is a promising drug target for heart defects caused by diabetes. A recent study has shown that PPARD induces glucose transport to the heart muscle cells and that diabetes patients have decreased PPARD expression in cardiac muscle when glycemic levels are high [29]. Synthetic agonists for PPARD have been developed for the treatment of obesity and related conditions, as overexpression increases glucose influx, reduces damage, improves insulin sensitivity, and reduces lipid accumulation [30].

In humans and mice, the PPARG gene has three splice variants: PPARG1, PPARG2, and PPARG3. PPARG1 and PPARG3 translate into identical proteins whereas PPARG2 contains an extra 28 amino acid regions in the NTD due to alternative splicing [31]. PPARG2 is an essential regulator of adipocyte differentiation and lipid storage in WAT, while PPARG1/3 has a more general function in lipid metabolism and is expressed in the colon and macrophages [32, 33]. Many synthetic agonists have also been designed for PPARG2, with the most well known being the TZDs and fibrates, both used for the treatment of metabolic disorders like type 2 diabetes mellitus (T2DM). TZDs that target PPARG are used to increase insulin sensitivity and reduce glycemia in the treatment of diabetes, and, additionally, PPARG activation has shown protective effects on the vasculature [34]. Activation of PPARG by TZDs has also been linked to increased weight gain in patients based on PPARG function in adipocyte differentiation and its involvement in lipid homeostasis [35]. As PPARG has also been shown to regulate certain processes in cancer development, it might be a possible additional target in the treatment of cancer. New generation drugs that target both PPARA and PPARG are very promising and have already been used in treatment, as they show hypolipidemic, hypotensive, anti-inflammatory, and antiatherogenic action [29, 36, 37].

## 2. Zebrafish as Model for Obesity

**Figure 1:**
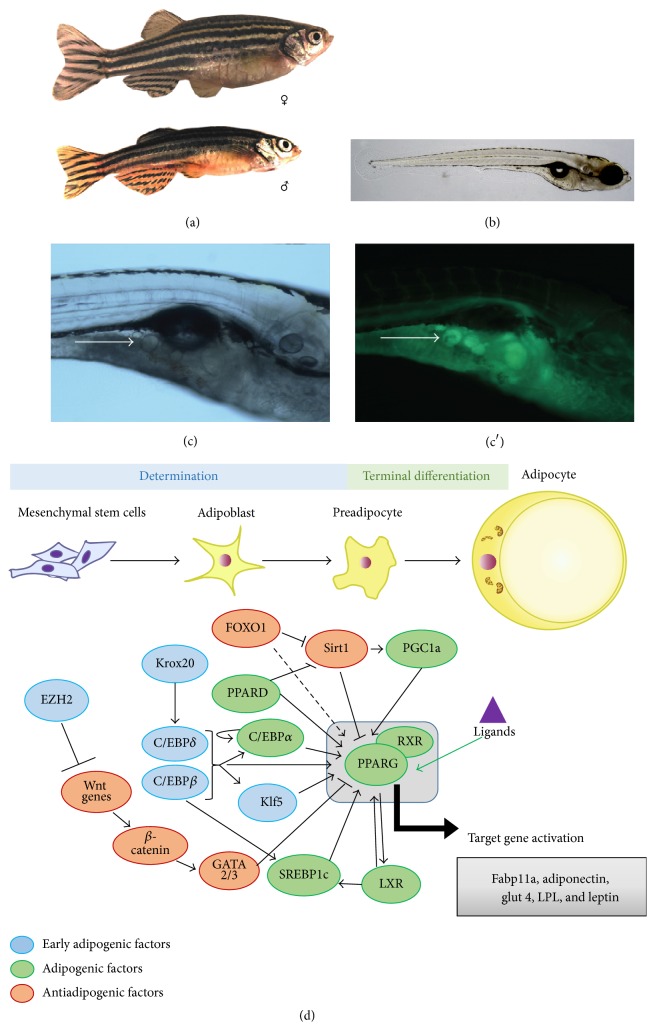
(a) An adult female and male zebrafish. (b) Developing zebrafish larvae (15 dpf). (c + c′) A transmission light image of adipocytes in developing larvae (15 dpf) (left) and a fluorescent image after staining lipids with LipidGreen (right). (d) Transcriptional network of factors important for adipocyte differentiation in zebrafish and mammals.

Numerous animal models have been used to study the etiology of obesity in order to gain better understanding of molecular mechanisms and possible treatments. In the last decade, zebrafish (*Danio rerio*), shown in adult and larval stages in Figure 1, have emerged as an excellent model to study human diseases [38] due to a number of advantages of this system. Zebrafish develop rapidly and have a short life cycle and detailed genome sequence information is available. The availability of new genome editing techniques like TALENs [39] and CRISPR-Cas9 [40], as well as several transgenesis tools (MultiSiteGateway Tol2, BAC transgenesis, Gal4/UAS, and Q system), enables the study of the function of genes. Zebrafish can also be used for high-throughput forward genetic and chemical screens [41–43], facilitating the identification of molecules that regulate biological functions.

Zebrafish are a promising model for obesity research, as lipid metabolism pathways are conserved between mammals and fish [44–46]. Zebrafish have the key organs that are important for energy homeostasis and metabolism in mammals, as well as other key functions such as appetite regulation in the brain [47], insulin regulation [48], endocrine signaling through leptin [49], and lipid storage in white adipocytes [50, 51]. Like humans, zebrafish kept on a high caloric diet show increased plasma triglyceride levels and hepatic steatosis as well as comparable expression patterns of genes involved in lipid metabolism such as LEP, SREBP, PPARA, PPARG, and NR3H1 [45]. It has also been shown that obesity in zebrafish coincides with an increased plasma fibrinogen concentration that is induced via IL-6 and IL-1Bl secreted from visceral white adipose tissue. Zebrafish, as well as mice, rats, and humans, also produce a higher amount of IL-6 and IL-1B upon high caloric feeding, adding to the evidence that major metabolic pathways between fish and mammals are very similar [45]. It is important to note, however, that zebrafish may not be an appropriate model for studying thermogenesis in higher vertebrates since brown adipose tissue has not been identified in poikilothermic animals. Zebrafish appear to not make thermogenic brown adipocytes, relying instead on thyroid hormone-mediated processes to generate heat in their muscles [52]. Although uncoupling proteins (UCPs 1–5) have been identified in zebrafish that are expressed in brain, liver, and muscle tissue but not in adipose tissue [53], differences in thermogenesis between mammals and fish are important to consider.

One major advantage of the zebrafish as a model of obesity is its optical transparence which allows temporal monitoring of adipocyte formation and fatty acid uptake* in vivo* [54]; adipocytes can be visualized in developing larvae with various dyes, including the sudanophilic dye Oil Red O (ORO) or Sudan dyes (Sudan III, Sudan IV, and Sudan Black B) and fluorescent dyes like Nile Red or LipidGreen (Figure 1(c)) [50, 55]. ORO and Sudan dyes are lipophilic (fat-soluble) dyes that can be applied as a soluble colorant for neutral lipids and cholesteryl esters. Nile Red binds to both neutral lipids and phospholipids while LipidGreen only binds to neutral lipids [55, 56]. Dyes such as Sudan Black B and ORO are fixative based dyes, and fixation techniques are time consuming and may also cause deformation of lipid droplets in tissue [57], while Nile Red and LipidGreen can be applied* in vivo*. The potential of using Nile Red staining in zebrafish larvae as a whole-organism test for screening pharmaceuticals and toxicological agents has recently been demonstrated [58].

## 3. Adipogenesis and Lipid Metabolism in Zebrafish

Adipose tissue develops from pluripotent mesenchymal stem cells (MSCs) and commitment to this cell lineage gives rise to preadipocytes (determination phase) and subsequent terminal differentiation adipocytes (differentiation phase) (Figure 1(d)) [59, 60]. In the transcriptional cascade leading to the differentiation of WAT (Figure 1(d)), the nuclear hormone receptor PPARG and CCAAT/enhancer-binding protein (C/EBP*α*) are key players. Two other members of the C/EBP family, C/EBP*β* and C/EBP*δ*, are also important factors during differentiation into adipocytes [59, 61, 62]. C/EBP*α* is a transcription factor that regulates PPARG expression but also autoregulates its own expression. In zebrafish, orthologs of these genes are expressed in adipocytes and liver [51]. The terminal differentiation markers like leptin and fabp11a are also expressed. Adiponectin has also been found in adult zebrafish adipose tissue [63].

During the first 4-5 days of development, zebrafish embryos are dependent on nutrients provided by the yolk sac which contains essential fat-soluble vitamins and triacylglycerol (TAG), as well as cholesterol. At days 5-6, the yolk sac is depleted and it is essential that larvae are provided with food to start to eat. The first signs of adipogenesis become visible at 8 days post fertilization (dpf) in the visceral cavity close to the pancreas but only in the minority of larvae. In most larvae, the first adipocytes translocate asymmetrically to the right visceral cavity [50]. Most adipocytes are observed from 12 dpf onwards (standard length (SL) of > 5 mm) in the pancreatic area [51]. In our laboratory, using LipidGreen staining, adipocytes are clearly visible at 15 dpf in the visceral region in a subset of larvae (Figure 1(c)). The amount of adipocytes is correlated with size of the larvae rather than age, suggesting body-length dependent lipid storage in adipocytes [50, 51]. At 17 dpf, all larvae have WAT in the pancreatic and visceral area, indicating that visceral WAT development is not dependent on size but regulated by age. Subcutaneous (20 dpf, SL > 8,2 mm) and cranial (22 dpf, SL > 9,4 mm) adipocytes develop in a size-dependent manner [51]. Adult fish have the largest adipocyte deposits in the visceral regions, but smaller deposits are found subcutaneously in the tail and jaw, and in the periorbital regions. Zebrafish adipocytes are metabolically active and are able to communicate via gap junctions [64]. The adipocyte lipid droplet size can vary between 1 and 100 *μ*m and while most contain one large droplet, some may contain multiple smaller droplets in the early stage of development. These characteristics are conformant to mammalian adipocyte development and provide evidence that zebrafish contain WAT similar to mammals [50, 65].

## 4. PPARs in Zebrafish

The presence of five ppar genes in the zebrafish genome has been described previously [80–82]. We performed an* in silico* study to examine the homology of zebrafish PPARs with orthologs in different species. Using the TBLASTX search tool in Ensembl database (Ensembl GRCz10, http://www.ensembl.org/Danio_rerio/Info/Index), we checked for all five* ppar* genes in the newest version of zebrafish genome sequence available (GRC10). Based on the Havana/Ensembl merged sequences and the mammalian/zebrafish protein sequences, cDNA, and Nucleotide Database (NCBI), transcripts could be identified (Table 2). The zebrafish genome has undergone complete genome duplication in the teleost lineage after the divergence of fish and mammal ancestors [83, 84]. Because of this, zebrafish have two PPARA (*pparaa*,* pparab*) and PPARD (*pparda*,* ppardb*) genes that are located on separate chromosomes (ohnologs). Only one PPARG (pparg) gene has been identified.* pparaa* is located on the reverse strand of chromosome 4 while* pparab* is located at forward strand of chromosome 25. The orthologs of PPARD are located on chromosome 22 (*pparda*) and chromosome 8 (*ppardb*) and are both positioned on the forward strand. In zebrafish,* pparg* gene is positioned on chromosome 11 on the reverse strand (Table 2). Comparing genomic regions of PPARs in human and zebrafish, we found conserved synteny regions using The Synteny Database [85].* pparaa* and* pparab* showed both synteny to the same region at human Chr 22 and showed synteny between their location on zebrafish genome. Also,* pparda* and* ppardb* have synteny to the same location at human Chr 6, and as their PPARA orthologs.* pparda* and* ppardb* showed synteny between their location on zebrafish genome. The zebrafish genome surrounding* pparg* is highly conserved compared to human Chr 3 (Supplemental data 3A–C in Supplementary Material available online at http://dx.doi.org/10.1155/2015/358029). The conserved regions contain the same genes in both organisms, which indicates a conserved functional relationship between syntenic genes.

**Figure 2:**
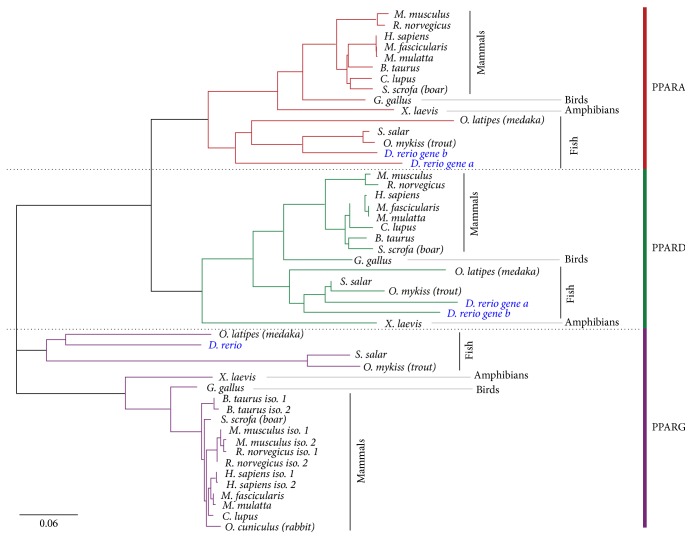
Phylogenetic tree of the PPAR orthologs in various organisms based on amino acid sequence difference (BLOSUM62 matrix). The phylogenetic tree is generated using the Geneious 9.0 software. Protein accession numbers information is provided in Supplemental Data 1.

Pparaa and Pparab encode for proteins that are 470 and 459 amino acids (aa) long, respectively. Pparda consists of 496 aa, and Ppardb is 517 aa long. Pparg is the longest PPAR in zebrafish and is 527 aa in size. Protein alignment and the phylogenetic tree are based on 49 amino acid sequences from 15 different species (Supplemental Data 1). The protein sequences were subjected to homology analyses using PRALINE multiple sequence alignment (http://www.ibi.vu.nl/programs/pralinewww/) (Supplemental Data 1 and Supplemental Data 2). The phylogenetic tree was constructed using the software Geneious 9.0 (BLOSUM62 matrix, Genetic Distance model: Jukes-Cantor; Biomatters, http://www.geneious.com/) (Figure 2). All zebrafish PPARs show protein identity to human and mice orthologs, with similarity of human PPARA to zebrafish Pparab and Pparaa of 74% and 67%, respectively. Zebrafish Pparda and Ppardb showed 71% and 73% similarity to human PPARD. Besides that, zebrafish Pparg is 67% similar to human PPARG (Table 3).

Amino acid sequence analysis of different homologues revealed that PPARs consist of a highly conserved protein family. The phylogenetic tree shows also that PPARA, PPARD, and PPARG proteins form distinct clusters of protein, although PPARA and PPARD were clustered together and the PPARG branch stands alone. The first gene duplication event of the PPAR gene family probably occurred in bony fish before being separated from birds and mammals. The second gene duplication is vertebrate specific and resulted in the different PPAR isotypes (A, D, and G) [86, 87]. After the gene duplication events, the PPARs acquired ligand binding capacities in an independent manner [88] and started to evolve by mutations in each PPARS resulting in refined specificity for ligands [87]. Zhao and colleagues [82] aligned the sequences of the DBD and LBD between human and 11 different species. The DBD and LBD for all PPAR are highly conserved between the different species, except for the DBD and LDB in Xenopus PPARD which are far much less conserved (83% and 75%, resp.). One explanation can be that PPARD is less evolved in Xenopus compared to the other species and therefore has a separated position in the phylogenetic tree.

The zebrafish PPAR proteins contain several large regions of amino acids that are highly conserved (Supplemental Data 2). The N-terminal region of zebrafish PPARs is less similar, as this region is highly variable between organisms. The N-terminal region contains the conserved AF-1 that can be activated by endogenous factors like hormones and cytokines. In contrast, the AF-2 site located in the C-terminal region remains well conserved. Like human PPARs, zebrafish Pparaa, Pparab, and Pparg contain a threonine/serine-rich NLS2 site that is involved in cytoplasm-nucleus shuttling of the PPARs. This site was not found in Ppard ohnologs, however, which have a threonine/serine-rich part earlier in the protein which could possibly result in a similar action. The overall conservation of PPARG is fairly high between organisms, except for three fish species including the Japanese medaka (*Oryzias latipes*), salmon (*Salmo salar*), and rainbow trout (*Oncorhynchus mykiss*), which have several (up to 5) additional amino acid regions throughout the protein. In all fish species, the LDB of PPARG is less conserved to human (74–78%) and this can be explained by the rapid evolutionary rate of PPARG in teleosts [82]. When comparing human and zebrafish PPARG, two major differences between zebrafish Pparg and human PPARG were found. Firstly, the first 30 amino acids from the N-terminus differ highly between zebrafish Pparg and both hPPARG isoforms. In this region of the protein, hPPARG2 contains extra 28 amino acids compared to the hPPARG1 isoform. Conversely, zebrafish Pparg contains only 16 amino acids in this region that differ in all except two amino acids from hPPARG2. Secondly, zebrafish Pparg has an extra 27 amino acid regions starting from the 156th amino acid. Other fish species also have extra amino acid sequence so it is possible that, during evolution, teleost fish gained additional coding sequences which can have implications for protein folding and functionality. We also observed that all fish species analysed have an extra protein sequence in the ligand binding domain visible at amino acid position 401 in the protein alignment. This extra sequence is not a part of the ligand binding pocket and presumably should not interfere with binding to specific ligands. However, a previous study has shown differences in ligand binding specificity, as sequence comparison of zebrafish PPARg to human and mice PPARG revealed residual differences within the LDB [89].

The phylogenetic tree shows that the zebrafish PPARA ohnologs have evolved further from each other compared to the zebrafish PPARD ohnologs (Figure 2). The zebrafish PPARD ohnologs have most likely retained the same function (subfunctionalization), while the zebrafish PPARA ohnologs might have been subject to neofunctionalization [80]. pparab has more similarities with salmon and rainbow trout, while pparaa is on a separate branch in the phylogenetic tree and has a higher homology with Japanese medaka. Additionally, zebrafish PPARA and PPARD ohnologs seem to be more related to each other than to zebrafish PPARG.

In fish, PPARs have been identified and characterized but not much is known about their expression during developmental stages and adipogenesis. Zebrafish PPARs are expressed during development and so far little is known of the expression profiles of the paralogs* pparaa*,* pparab*,* pparda*, and* ppardb*. The limited expression studies that have been performed show that* ppardb* and* pparg* mRNA are already expressed early in development (5–10 hours post fertilization (hpf)) in whole embryo. From 20 hpf on, the expression is more intensive in the head region and continues to be specifically expressed in the head, branchial arches, and pectoral fin at 36 hpf. From this time point, expression data for* pparda* is also available and comparable to that of* ppardb* and* pparg.* At 5 dpf, gene expression is detected around the swim bladder (*pparda*,* ppardb*, and* pparg*), in the liver (*pparda*,* pparg*), in the intestinal bulb (*pparda*), and also in the complete intestinal tract (*pparg*) [80]. The expression patterns of* ppard* paralogs and* pparg* can be an indication that they play a role in adipogenesis since it is known that first adipocytes arise in the pancreatic region underneath the swim bladder [50, 51]. During the larval stage (15 dpf),* pparg* mRNA is localized in developing adipocytes within the pancreas and the intestinal epithelium in the same cells where Nile Red staining was observed [50]. Expression of the* pparg* gene is specifically detected in visceral adipose tissue and pancreas as opposed to liver [51].

## 5. Conclusions

PPARs are important factors in energy homeostasis and obesity development. While PPARA and PPARD are necessary for lipid breakdown, PPARG plays a role in lipid accumulation and adipogenesis. Single nucleotide polymorphisms in PPARs have been implicated in the development of obesity, T2DM, lipodystrophy, dyslipidemias, and cardiovascular risk. The zebrafish is an emerging model for research in obesity and related metabolic diseases and could be very useful for providing insight into the role of PPARs in the origins of obesity. All five PPAR genes (*pparaa*,* pparab*,* pparda*,* ppardb*, and* pparg*) show protein similarity to human and mice PPARs varying from 67 to 74%. Three of the five zebrafish PPARs (*pparda*,* ppardb*, and* pparg*) show expression of mRNA in regions around swim bladder and pancreas that correlate to the sites where first adipocytes will develop, and at 15 dpf,* pparg* expression is detected in developing adipocytes. This colocalization suggests a role for PPARs in adipogenesis. However, detailed expression studies of PPARs during early stages of adipocyte development are not available, and to determine the subcellular expression of the different PPARs in zebrafish, specific antibody staining needs to be performed or transgenic fish lines should be generated. In addition, more conclusive evidence of the function of PPAR in zebrafish adipogenesis can be obtained by the generation of mutant lines. Taken together, this review shows that the zebrafish is a promising model for elucidating the specific functions of PPARs in adipogenesis and obesity.

## Supplementary Material

In Table S1, we provide all the PPAR protein Accession numbers used for protein alignment and the phylogenetic tree. In Supplemental data 2 A-C, we present the protein alignment of PPARA, PPARD, and PPARG. Supplemental data 3 A-C shows the synteny between zebrafish genomic regions to the human genome for the different PPARs.

## Figures and Tables

**Table 1:** Endogenous and synthetic ligands of vertebrate PPARs.

PPAR	Ligand type	Potential agonists
PPARA	Endogenous	Fatty acids (lauric acid, linoleic acid, linolenic acid, arachidonic acid, eicosapentaenoic acid (*Xenopus*), docosahexaenoic acid (*Xenopus*), petroselinic acid (*Xenopus*), oleic acid (*Xenopus*), and elaidic acid (*Xenopus*)) [66, 67] Eicosanoids (fatty acid-derived) (e.g., leukotriene B_4_) [68, 69] Prostaglandin J2 (fatty acid-derived) [70] Endocannabinoids [71]
	Synthetic	Nonsteroidal anti-inflammatory drugs (NSAIDs) [72] Fibrates (e.g., gemfibrozil, Bezafibrate, clofibrate, fenofibrate, ciprofibrate, pirinixic acid (Wy 14643), and GW2331) [3, 67, 73, 74] ETYA (5,8,11,14-eicosatetraynoic acid) (*Xenopus*) [66]

PPARD	Endogenous	Fatty acids (e.g., docosahexaenoic acid, linoleic acid) [69] Very Low Density Lipoproteins (VLDL) components [75]
	Synthetic	Fibrates (Wy-14,643, Bezafibrate) [69] GW501516, GW800644 [76, 77]

PPARG	Endogenous	Fatty acids (docosahexaenoic acid, linoleic acid (mouse)) [14, 69] Prostaglandin J2 metabolite 15-deoxy-delta 12,14-PGJ2 [70, 78]
	Synthetic	NSAIDs [72] Thiazolidinediones [79] ETYA (5,8,11,14-eicosatetraynoic acid) (mouse) [14] Prostaglandin J2 derivatives [70, 78] Fibrates (Bezafibrate, clofibrate, and GW2331) [14, 67]

**Table 2:** Location and characteristics of the PPAR genes in zebrafish genome (GRCz10, Havana, and NCBI).

PPAR	Gene	Chromosome location	GRCz10	NCBI Nucleotide	cds (bp)	Protein (aa)	Exons
*pparaa*	ENDARG00000031777	4; rev	CM002888.1	NM_001161333	1,413	470	6
*pparab*	ENDARG00000054323	25; forw	CM002909.1	NM_001102567	1,380	459	7
*pparda*	ENDARG00000044525	22; forw	CM002906.1	XM_694808, XM005168286	1491	496	7
*ppardb*	ENDARG00000009473	8; forw	CM002892.1	NM_131468	1554	517	7
*pparg*	ENDARG00000031848	11; rev	CM002895.1	NM_131467	1584	527	7

**Table 3:** Comparison of PPARs between human, mouse, and zebrafish.

PPAR	Human	Mouse	Zebrafish
PPARA			
Genes	1	1	2
Splice variants	1	1	1 per gene
Isoforms	1	1	1 per gene
Size (cds, bp)	1,407	1,407	*pparaa*: 1,413 *pparab*: 1,380
Protein similarity to zebrafish	67% for *pparaa* 74% for *pparab*	65% for *pparaa* 71% for *pparab*	

PPARD			
Genes	1	1	2
Splice variants	1	1	1 per gene
Isoforms	1	1	1 per gene
Size (cds, bp)	1,326	1,323	*pparda*: 1,491 *ppardb*: 1,554
Protein similarity to zebrafish	71% for *pparda* 73% for *ppardb*	70% for *pparda* 73% for *ppardb*	

PPARG			
Genes	1	1	1
Splice variants	4	1	1
Isoforms	2	2	1
Size (cds, bp)	PPARG1 = 1,434 PPARG2 = 1,518	PPARG1 = 1,428 PPARG2 = 1,518	*pparg*: 1,584
Protein similarity to zebrafish	67%	67%	
