# Supplementary material for: Zebrafish as a Model to Study the Role of Peroxisome Proliferating-Activated Receptors in Adipogenesis and Obesity
Source: PPAR Res. 2015 Nov 30;2015:358029. doi: 10.1155/2015/358029 (PMC4677228; doi:10.1155/2015/358029)
Supplement: Supplementary file 1 — In Table S1, we provide all the PPAR protein Accession numbers used for protein alignment and the phylogenetic tree. In Supplemental data 2 A-C, we present the protein alignment of PPARA, PPARD, and PPARG. Supplemental data 3 A-C shows the synteny between zebrafish genomic regions to the human genome for the different PPARs. [file 358029.f1.pdf]

Supplemental data 1: Accesion numbers for PPAR proteins

Table S1: All PPAR protein Accession numbers used for Protein alignment using Praline and Phylogentic tree (Geneious)

PPAR	Species		Accession numbers
PPARA	Mouse	*Mus musculus*	NP_001106889.1
	Rat	*Rattus norvegicus*	NP_037328.1
	Human	*Homo sapiens*	CAG33716.1
	Crab-eating macaque	*Macaca fascicularis*	XP_005567059.1
	Rhesus macaque	*Macaca mulatta*	NP_001028201.1
	Chicken	*Gallus gallus*	NP_001001464.1
	African clawed frog	*Xenopus laevis*	AAH79684.1
	Japanese medaka	*Oryzias latipes*	BAH58103.1
	Cow	*Bos taurus*	NP_001029208.1
	Salmon	*Salmo salar*	NP_001117032.1
	Dog	*Canis lupus familiaris*	NP_001003093.1
	Wild boar	*Sus scrofa*	NP_001037991.1
	Rainbow trout	*Oncorhynchus mykiss*	ADN92684.1
	Zebrafish a	*Danio rerio*	NP_001154805.1
	Zebrafish b		NP_001096037.1
PPARD	Mouse	*Mus musculus*	NP_035275.1
	Rat	*Rattus norvegicus*	NP_037273.2
	Human	*Homo sapiens*	NP_001165289.1
	Crab-eating macaque	*Macaca fascicularis*	XP_005553282.1
	Rhesus macaque	*Macaca mulatta*	AFI33297.1
	Chicken	*Gallus gallus*	NP_990059.1
	African clawed frog	*Xenopus laevis*	NP_001081310.1
	Japanese medaka	*Oryzias latipes*	BAH58104.1
	Cow	*Bos taurus*	NP_001077105.1
	Salmon	*Salmo salar*	AAK76399.1
	Dog	*Canis lupus familiaris*	NP_001041567.1
	Wild boar	*Sus scrofa*	NP_999317.2
	Rainbow trout	*Oncorhynchus mykiss*	ADN92685.1
	Zebrafish a	*Danio rerio*	XP_005168343.1
	Zebrafish b		NP_571543.1
PPARG	Mouse 1	*Mus musculus*	NP_001120802.1
	Mouse 2		NP_035276.2
	Rat 1	*Rattus norvegicus*	NP_037256.1
	Rat 2		NP_001138838.1
	Human 1	*Homo sapiens*	NP_005028.4
	Human 2		NP_056953.2
	Crab-eating macaque	*Macaca fascicularis*	NP_001270028.1
	Rhesus macaque	*Macaca mulatta*	NP_001028032.1
	Chicken	*Gallus gallus*	NP_001001460.1
	African clawed frog	*Xenopus laevis*	NP_001081312.1
	Japanese medaka	*Oryzias latipes*	NP_001158348.1
	Cow 1	*Bos taurus*	AAI16099.1
	Cow 2		NP_851367.1
	Salmon	*Salmo salar*	CAC95231.1
	Dog	*Canis lupus familiaris*	NP_001019803.1
	Wild boar	*Sus scrofa*	NP_999544.1
	Rabbit	*Oryctolagus cuniculus*	NP_001075617.1
	Rainbow trout	*Oncorhynchus mykiss*	ADN92686.1
	Zebrafish	*Danio rerio*	NP_571542.1

Supplemental data 2A: Protein alignment PPARA

```
                     10         20         30         40         50
mmPPARA     ----MVDTES PICPLSPLEA DDLESPLSEE FLQEMGNIQE ISQSIGEESS
rnPPARA     ----MVDTES PICPLSPLEA DDLESPLSEE FLQEMGNIQE ISQSLGEESS
btPPARA     --MEMVDTES PIGPLSPLEA DDLESPLSAD FLQEMGTIQE ISQSIGEDSS
clPPARA     ----MVDTES PICPLSPLEA DDLESPLSEE FLQEMGNIQE ISQSIGEDSS
ssPPARA     ----MVDTES PICPLSPLEA DDLESPLSEE FLQEMGTIQE ISQSIGEDSS
hsPPARA     ----MVDTES PLCPLSPLEA GDLESPLSEE FLQEMGNIQE ISQSIGEDSS
ggPPARA     ----MVDTEN QLYPLTPLEE DDIGSPLSGE FLQDMENIQD ISQSLGDDSS
xlPPARA     MSTIMVDTNS ELCILTPLDE DDLESPLSGE FLQDIVDIQD ITQTIGDDGS
macfPPARA   ----MVDTES PLCPLSPLEA GDLESPLSEE FLQEMGNIQE ISQSIGEDSS
macmPPARA   ----MVDTES PLCPLSPLEA GDLESPLSEE FLQEMGNIQE ISQSIGEDSS
drPPARAa    ----MVDMPS LYSPSSPLGD PIMYSPLSGE LIGDMQVLED ISQSLSDDTF
olPPARA     ----M--AAE LYSPPSPLGD SLLDSPLCGD LIEDL---RK ISASIG-DNS
drPPARAb    ----MVDMEN RYRPPSPLDD SVLDS---AL FVRGMEELRD ISQSMDEDAL
omPPARA     ----M---AR XYRPPSPLED SVLGSPVCGD FLGGMEELQD IPQSIDGDTL
salsPPARA   ----M---AS HYRPPSPLED SVLGSPVCGD FLGGMEELQD ISQSIDGDAL
Consistency 0000*66567 464858**75 5595*87657 8866944787 *899875976

                     60         70         80         90        100
mmPPARA     GSFGFADYQY LGSCPGSEGS V---ITDTLS PASSPSSVSC PVIPASTDES
rnPPARA     GSFSFADYQY LGSCPGSEGS V---ITDTLS PASSPSSVSC PAVPTSTDES
btPPARA     GSFSFTEYQY LGSGPGSDGS V---ITDTLS PASSPSSVSY PAVPGSAEES
clPPARA     GSFSFTEYQY LGSGPGSDGS V---ITDTLS PAPSPSSVTH PAAPGGAEEP
ssPPARA     GSFSFTDYQY LGSGPGSDGS V---ITDTLS PASSPSSVTY PVAPAGADES
hsPPARA     GSFGFTEYQY LGSCPGSDGS V---ITDTLS PASSPSSVTY PVVPGSVDES
ggPPARA     GALSLTEFQS LGNGPGSDGS V---ITDTLS PASSPSSINF ATAPGSIDES
xlPPARA     TPFGASEHQF FGNSPGSIGS VSTDLTDTLS PASSPASITF PAASGSAEDA
macfPPARA   GSFGFTEYQY LGSCPGSDGS V---ITDTLS PASSPSSVTY PVVPGSVDES
macmPPARA   GSFSFTEYQY LGSCPGSDGS V---ITDTLS PASSPSSVTY PVVPGSVDES
drPPARAa    NSFHMLDYQN CDTAVDNSS- ----ILDVLT PASSPSSEVF -SASTGQDEN
olPPARA     LEFDFPECQR ND--SGAKSS V---MPDPLT PASSPSPEVC GAAPA--AED
drPPARAb    SSFEMTENQS -GLGSGSESS T---ELDALT PASSPSSGVY G-CPVGQDEF
omPPARA     SSLDVPEYLS QSS-NGSEGS T---VLDALT PASSPSSGGY G-AAAGLEEF
salsPPARA   SSLDVPEYQS QSS-NGSEGS T---VLDALT PASSPSSGGY G-AAAGLEEF
Consistency 5785658685 4762598688 700076*6*8 **9**99555 5467564795

                    110        120        130        140        150
mmPPARA     PGSALNIECR ICGDKASGYH YGVHACEGCK GFFRRTIRLK LVYDKCDRSC
rnPPARA     PGNALNIECR ICGDKASGYH YGVHACEGCK GFFRRTIRLK LAYDKCDRSC
btPPARA     SSIALNIECR ICGDKASGYH YGVHACEGCK GFFRRTIRLK LVYDKCDRSC
clPPARA     SSVALNIECR ICGDRASGYH YGVHACEGCK GFFRRTIRLK LAYDKCDRSC
ssPPARA     PSVALNIECR ICGDKASGYH YGVHACEGCK GFFRRTIRLK LVYDKCDRSC
hsPPARA     PSGALNIECR ICGDKASGYH YGVHACEGCK GFFRRTIRLK LVYDKCDRSC
ggPPARA     PSGAENIECR ICGDKASGYH YGVHACEGCK GFFRRTIRLK LIYDKCDRNC
xlPPARA     ACKSLNLECR VCSDKASGFH YGVHACEGCK GFFRRTIRLK LVYDRCERMC
macfPPARA   PSGALNIECR ICGDKASGYH YGVHACEGCK GFFRRTIRLK LVYDKCDRSC
macmPPARA   PSGALNIECR ICGDKASGYH YGVHACEGCK GFFRRTIRLK LVYDKCDRSC
drPPARAa    SSGSLTLECR VCADRASGFH YGVHACEGCK GFFRRTIRLK LEYDKCERNC
olPPARA     SVGPLSLECR VCSDRASGFH YGVHACEGCK GFFRRTIRLK LEYNKCERNC
drPPARAb    TSTSLNLECR VCSDRASGYH YGVHACEGCK GFFRRTIRLK LEYDKCERRC
omPPARA     SSTSLNLECR VCADRASGYH YGVHACEGCK GFFRRTIRLK LEYDKCERHC
salsPPARA   SSTSLNLECR VCADRASGYH YGVHACEGCK GFFRRTIRLK LEYDKCERRC
Consistency 5647888*** 9*6*8***8* ********** ********** *5*99*8*5*

                    160        170        180        190        200
mmPPARA     KIQKKNRNKC QYCRFHKCLS VGMSHNAIRF GRMPRSEKAK LKAEILTCEH
rnPPARA     KIQKKNRNKC QYCRFHKCLS VGMSHNAIRF GRMPRSEKAK LKAEILTCEH
btPPARA     KIQKKNRNKC QYCRFHKCLS VGMSHNAIRF GRMPRSEKAK LKAEILTCEH
clPPARA     KIQKKNRNKC QYCRFHKCLS VGMSHNAIRF GRMPRSEKAK LKAEILTCEQ
ssPPARA     KIQKKNRNKC QYCRFHKCLS AGMSHNAIRF GRMPRSEKAK LKAEILTCEH
hsPPARA     KIQKKNRNKC QYCRFHKCLS VGMSHNAIRF GRMPRSEKAK LKAEILTCEH
ggPPARA     KIQKKNRNKC QYCRFQKCLS VGMSHNAIRF GRMPRSEKAK LKAEILTGEN
xlPPARA     KIQKKNRNKC QYCRFEKCLN VGMSHNAIRF GRMPRSEKAK LKAEVLMCDQ
macfPPARA   KIQKKNRNKC QYCRFHKCLS VGMSHNAIRF GRMPRSEKAK LKAEILTCEH
macmPPARA   KIQKKNRNKC QYCRFHKCLS VGMSHNAIRF GRMPRSEKAK LKAEILTCEH
drPPARAa    KIQKKNRNKC QYCRFRKCLA VGMSHNAIRF GRIPQSEKQR LKAEQDVSGK
olPPARA     KIQKKNRNKC QYCRFNKCMI VGMSHNAIRF GRMPHAEKLK LKEESKVAEK
drPPARAb    KIQKKNRNKC QYCRFQKCLS VGMSHNAIRF GRMPQSEKLR LKAEILTGER
omPPARA     KIQKKNRNKC QYCRFQKCLS VGMSHNAIRF GRMPQSEKLK LKAEILTGDR
salsPPARA   KIQKKNRNKC QYCRFQKCLS VGMSHNAIRF GRMPQSEKLK LKAEILTGDR
Consistency ********** *****6**97 9********* **9*79**69 **9*777575
```

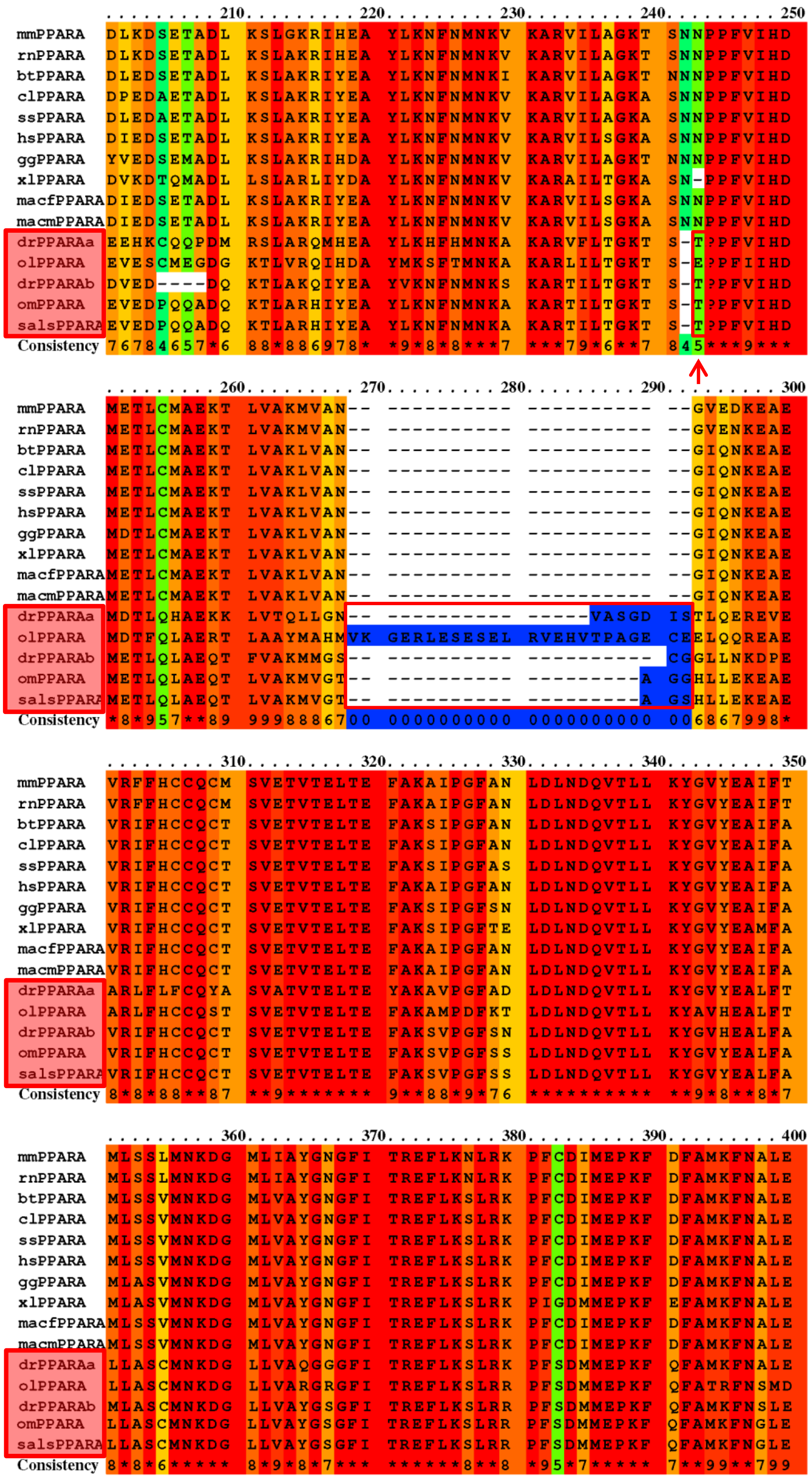

. . . . 210. . . . . 220. . . . . 230. . . . . 240. 250
mmPPARA DLKDSETADL KSLGKRIHEA YLKNFNMNKV KARVILAGKT SNNPPFVIHD
rnPPARA DLKDSETADL KSLAKRIHEA YLKNFNMNKV KARVILAGKT SNNPPFVIHD
btPPARA DLEDSETADL KSLAKRIYEA YLKNFNMNKI KARVILAGKT NNNPPFVIHD
clPPARA DPEDAETADL KSLAKRIYEA YLKNFNMNKV KARVILAGKA SNNPPFVIHD
ssPPARA DLEDAETADL KSLAKRIYEA YLKNFNMNKV KARVILAGKA SNNPPFVIHD
hsPPARA DIEDSETADL KSLAKRIYEA YLKNFNMNKV KARVILSGKA SNNPPFVIHD
ggPPARA YVEDSEMADL KSLAKRIHDA YLKNFNMNKV KARVILAGKT NNNPPFVIHD
xlPPARA DVKDTQMADL LSLARLIYDA YLKNFNMNKV KARAILTGKA SN-PPFVIHD
macfPPARA DIEDSETADL KSLAKRIYEA YLKNFNMNKV KARVILSGKA SNNPPFVIHD
macmPPARA DIEDSETADL KSLAKRIYEA YLKNFNMNKV KARVILSGKA SNNPPFVIHD
drPPARAa EEHKCQQPDM RSLARQMHEA YLKHFHMNKA KARVFLTGKT S-TPPFVIHD
olPPARA EVESCMEGDG KTLVRQIHDA YMKSFTMNKA KARLILTGKT S-EPPFIIHD
drPPARAb DVED----DQ KTLAKQIYEA YVKNFNMNKS KARTILTGKT S-TPPFVIHD
omPPARA EVEDPQQADQ KTLARHIYEA YLKNFNMNKA KARTILTGKT S-TPPFVIHD
salsPPARA EVEDPQQADQ KTLARHIYEA YLKNFNMNKA KARTILTGKT S-TPPFVIHD
Consistency 7678465 7*6 88*886978* *9*8*8***7 ***79*6**7 845***9***
. . . . 260. . . . . 270. . . . . 280. . . . . 290. 300
mmPPARA METLCMAEKT LVAKMVAN-- ---------- ---------- --GVEDKEAE
rnPPARA METLCMAEKT LVAKMVAN-- ---------- ---------- --GVENKEAE
btPPARA METLCMAEKT LVAKLVAN-- ---------- ---------- --GIQNKEAE
clPPARA METLCMAEKT LVAKLVAN-- ---------- ---------- --GIQNKEAE
ssPPARA METLCMAEKT LVAKLVAN-- ---------- ---------- --GIQNKEAE
hsPPARA METLCMAEKT LVAKLVAN-- ---------- ---------- --GIQNKEAE
ggPPARA MDTLCMAEKT LVAKLVAN-- ---------- ---------- --GIQNKEAE
xlPPARA METLCMAEKT LVAKLVAN-- ---------- ---------- --GIQNKEAE
macfPPARA METLCMAEKT LVAKLVAN-- ---------- ---------- --GIQNKEAE
macmPPARA METLCMAEKT LVAKLVAN-- ---------- ---------- --GIQNKEAE
drPPARAa MDTLQHAEKK LVTQLLGN-- ---------- -----VASGD ISTLQEREVE
olPPARA MDTFQLAERT LAAYMAHMVK GERLESESEL RVEHVTPAGE CEELQQREAE
drPPARAb METLQLAEQT FVAKMMGS-- ---------- ---------- CGGLLNKDPE
omPPARA METLQLAEQT LVAKMVGT-- ---------- ---------A GGHLLEKEAE
salsPPARA METLQLAEQT LVAKMVGT-- ---------- ---------A GSHLLEKEAE
Consistency *8*957**89 9998886700 0000000000 0000000000 006867998*
. . . . 310. . . . . 320. . . . . 330. . . . . 340. 350
mmPPARA VRFFHCCQCM SVETVTELTE FAKAIPGFAN LDLNDQVTLL KYGVYEAIFT
rnPPARA VRFFHCCQCM SVETVTELTE FAKAIPGFAN LDLNDQVTLL KYGVYEAIFT
btPPARA VRIFHCCQCT SVETVTELTE FAKSIPGFAN LDLNDQVTLL KYGVYEAIFA
clPPARA VRIFHCCQCT SVETVTELTE FAKSIPGFAN LDLNDQVTLL KYGVYEAIFA
ssPPARA VRIFHCCQCT SVETVTELTE FAKSIPGFAS LDLNDQVTLL KYGVYEAIFA
hsPPARA VRIFHCCQCT SVETVTELTE FAKAIPGFAN LDLNDQVTLL KYGVYEAIFA
ggPPARA VRIFHCCQCT SVETVTELTE FAKSIPGFSN LDLNDQVTLL KYGVYEAIFA
xlPPARA VRIFHCCQCT SVETVTELTE FAKSIPGFTE LDLNDQVTLL KYGVYEAMFA
macfPPARA VRIFHCCQCT SVETVTELTE FAKAIPGFAN LDLNDQVTLL KYGVYEAIFA
macmPPARA VRIFHCCQCT SVETVTELTE FAKAIPGFAN LDLNDQVTLL KYGVYEAIFA
drPPARAa ARLFLFCQYA SVATVTELTE YAKAVPGFAD LDLNDQVTLL KYGVYEALFT
olPPARA ARLFHCCQST SVETVTELTE FAKAMPDFKT LDLNDQVTLL KYAVHEALFT
drPPARAb VRIFHCCQCT SVETVTELTE FAKSVPGFSN LDLNDQVTLL KYGVHEALFA
omPPARA VRIFHCCQCT SVETVTELTE FAKSVPGFSS LDLNDQVTLL KYGVYEALFA
salsPPARA VRIFHCCQCT SVETVTELTE FAKSVPGFSS LDLNDQVTLL KYGVYEALFA
Consistency 8*8*88**87 **9******* 9**88*9*76 ********** **9*8**8*7
. . . . 360. . . . . 370. . . . . 380. . . . . 390. 400
mmPPARA MLSSLMNKDG MLIAYGNGFI TREFLKNLRK PFCDIMEPKF DFAMKFNALE
rnPPARA MLSSLMNKDG MLIAYGNGFI TREFLKNLRK PFCDIMEPKF DFAMKFNALE
btPPARA MLSSVMNKDG MLVAYGNGFI TREFLKSLRK PFCDIMEPKF DFAMKFNALE
clPPARA MLSSVMNKDG MLVAYGNGFI TREFLKSLRK PFCDIMEPKF DFAMKFNALE
ssPPARA MLSSVMNKDG MLVAYGNGFI TREFLKSLRK PFCDIMEPKF DFAMKFNALE
hsPPARA MLSSVMNKDG MLVAYGNGFI TREFLKSLRK PFCDIMEPKF DFAMKFNALE
ggPPARA MLASVMNKDG MLVAYGNGFI TREFLKSLRK PFCDIMEPKF DFAMKFNALE
xlPPARA MLASVMNKDG MLVAYGNGFI TREFLKSLRK PIGDMMEPKF EFAMKFNALE
macfPPARA MLSSVMNKDG MLVAYGNGFI TREFLKSLRK PFCDIMEPKF DFAMKFNALE
macmPPARA MLSSVMNKDG MLVAYGNGFI TREFLKSLRK PFCDIMEPKF DFAMKFNALE
drPPARAa LLASCMNKDG LLVAQGGGFI TREFLKSLRK PFSDMMEPKF QFAMKFNALE
olPPARA LLASCMNKDG LLVARGRGFI TREFLKSLRR PFSDMMEPKF QFATRFNSMD
drPPARAb MLASCMNKDG LLVAYGSGFI TREFLKSLRR PFSDMMEPKF QFAMKFNSLE
omPPARA LLASCMNKDG LLVAYGSGFI TREFLKSLRR PFSDMMEPKF QFAMKFNGLE
salsPPARA LLASCMNKDG LLVAYGSGFI TREFLKSLRR PFSDMMEPKF QFAMKFNGLE
Consistency 8*8*6***** 8*9*8*7*** ******8**8 *95*7***** 7**99**799

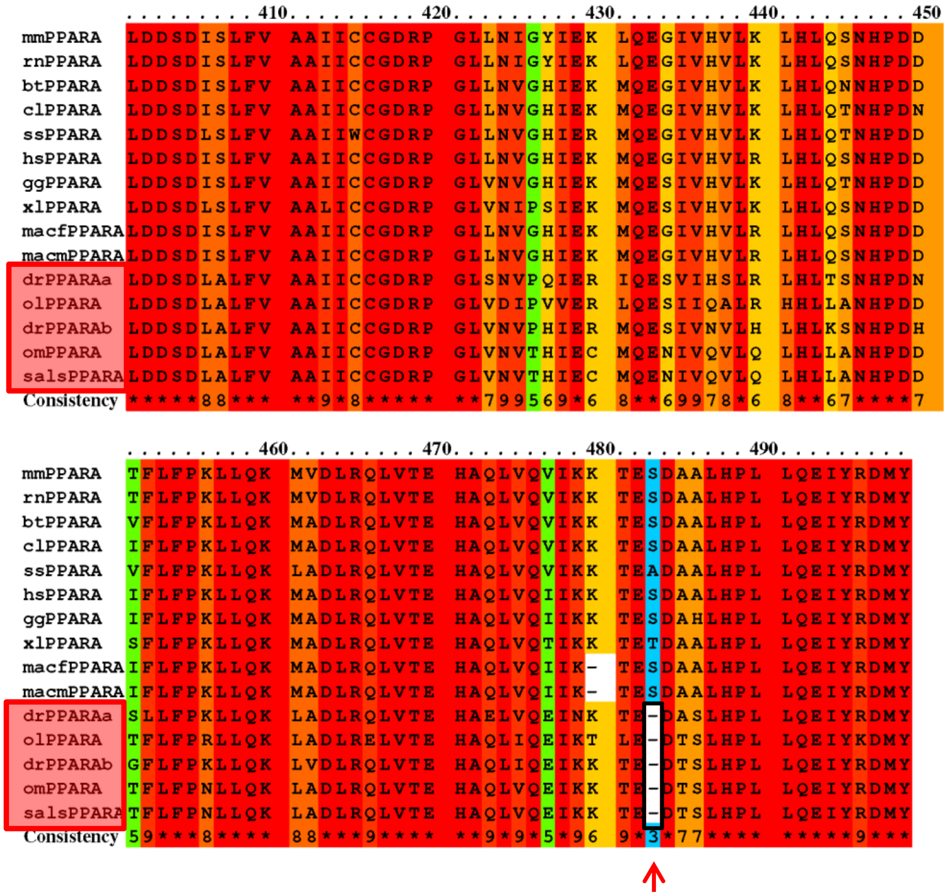

. . . . 410. . . . . 420. . . . . 430. . . . . 440. 450
mmPPARA LDDSDISLFV AAIICCGDRP GLLNIGYIEK LQEGIVHVLK LHLQSNHPDD
rnPPARA LDDSDISLFV AAIICCGDRP GLLNIGYIEK LQEGIVHVLK LHLQSNHPDD
btPPARA LDDSDISLFV AAIICCGDRP GLLNVGHIEK MQEGIVHVLK LHLQNNHPDD
clPPARA LDDSDISLFV AAIICCGDRP GLLNVGHIEK MQEGIVHVLK LHLQTNHPDN
ssPPARA LDDSDLSLFV AAIIWCGDRP GLLNVGHIER MQEGIVHVLK LHLQTNHPDD
hsPPARA LDDSDISLFV AAIICCGDRP GLLNVGHIEK MQEGIVHVLR LHLQSNHPDD
ggPPARA LDDSDISLFV AAIICCGDRP GLVNVGHIEK MQESIVHVLK LHLQTNHPDD
xlPPARA LDDSDLSLFV AALICCGDRP GLVNIPSIEK MQESIVHVLK LHLQSNHPDD
macfPPARA LDDSDISLFV AAIICCGDRP GLLNVGHIEK MQEGIVHVLR LHLQSNHPDD
macmPPARA LDDSDISLFV AAIICCGDRP GLLNVGHIEK MQEGIVHVLR LHLQSNHPDD
drPPARAa LDDSDLALFV AAIICCGDRP GLSNVPQIER IQESVIHSLR LHLTSNHPDN
olPPARA LDDSDLALFV AAIICCGDRP GLVDIPVVER LQESIIQALR HHLLANHPDD
drPPARAb LDDSDLALFV AAIICCGDRP GLVNVPHIER MQESIVNVLH LHLKSNHPDH
omPPARA LDDSDLALFV AAIICCGDRP GLVNVTHIEC MQENIVQVLQ LHLLANHPDD
salsPPARA LDDSDLALFV AAIICCGDRP GLVNVTHIEC MQENIVQVLQ LHLLANHPDD
Consistency *****88*** **9*8***** **799569*6 8**69978*6 8**67****7
. . . . 460. . . . . 470. . . . . 480. . . . . 490. . . . .
mmPPARA TFLFPKLLQK MVDLRQLVTE HAQLVQVIKK TESDAALHPL LQEIYRDMY
rnPPARA TFLFPKLLQK MVDLRQLVTE HAQLVQVIKK TESDAALHPL LQEIYRDMY
btPPARA VFLFPKLLQK MADLRQLVTE HAQLVQVIKK TESDAALHPL LQEIYRDMY
clPPARA IFLFPKLLQK MADLRQLVTE HAQLVQVIKK TESDAALHPL LQEIYRDMY
ssPPARA VFLFPKLLQK LADLRQLVTE HAQLVQVIKK TEADAALHPL LQEIYRDMY
hsPPARA IFLFPKLLQK MADLRQLVTE HAQLVQIIKK TESDAALHPL LQEIYRDMY
ggPPARA IFLFPKLLQK MADLRQLVTE HAQLVQIIKK TESDAHLHPL LQEIYRDMY
xlPPARA SFLFPKLLQK MADLRQLVTE HAQLVQTIKK TETDAALHPL LQEIYRDMY
macfPPARA IFLFPKLLQK MADLRQLVTE HAQLVQIIK- TESDAALHPL LQEIYRDMY
macmPPARA IFLFPKLLQK MADLRQLVTE HAQLVQIIK- TESDAALHPL LQEIYRDMY
drPPARAa SLLFPKLLQK LADLRQLVTE HAELVQEINK TE-DASLHPL LQEIYRDMY
olPPARA TFLFPRLLQK LADLRELVTE HAQLIQEIKT LE-DTSLHPL LQEIYKDMY
drPPARAb GFLFPKLLQK LVDLRQLVTE HAQLIQEIKK TE-DTSLHPL LQEIYRDMY
omPPARA TFLFPNLLQK LADLRQLVTE HAQLVQEIKK TE-DTSLHPL LQEIYRDMY
salsPPARA TFLFPNLLQK LADLRQLVTE HAQLVQEIKK TE-DTSLHPL LQEIYRDMY
Consistency 59***8**** 88***9**** **9*9*5*96 9*3*77**** *****9***

Supplemental data 2B: Protein alignment PPARD

Unconserved 0 1 2 3 4 5 6 7 8 9 10 Conserved

```
           .........10 .........20 .........30 .........40 .........50
mmPPARD    ---------- ---------- ---------- ---------- ----------
rnPPARD    ---------- ---------- ---------- ---------- ----------
ggPPARD    ---------- ---------- ---------- ---------- ----------
hsPPARD    ---------- ---------- ---------- ---------- ----------
macfPPARD  ---------- ---------- ---------- ---------- ----------
macmPPARD  ---------- ---------- ---------- ---------- ----------
btPPARD    ---------- ---------- ---------- ---------- ----------
ssPPARD    ---------- ---------- ---------- ---------- ----------
clPPARD    ---------- ---------- ---------- ---------- ----------
omPPARD    ---------- ---------- ---------- ---------- ----------
olPPARD    MDGFQHSAAK QP--EGVNGY FEP-ASPQDT ADVRWKHPEG -E--------
drPPARDb   MEQVEEPTSE MKCDGQVDGV MKS-QSSLQT ELSSVPILRD -AAWLGAQEG
drPPARDa   ---------- -------MSV CEMDRRHVLA LRSRLPRNLG SVGGVYM-DP
xlPPARD    ---------- ---------- ---------- ---------- ----------
salsPPARD  ---------- ---------- ---------- ---------- ----------
Consistency 0000000000 0000000000 0000000000 0000000000 0000000000

           .........60 .........70 .........80 .........90 ........100
mmPPARD    ---------- -MEQPQE--- ETPEAREEEK E-----EVAM GDG-------
rnPPARD    ---------- -MEQPQE--- ETPEAREEEK E-----EVAT GDG-------
ggPPARD    ---------- -MEQLQEEVP EVREEEEEEE E-----AVTV PSG-------
hsPPARD    ---------- -MEQPQEE-- APEVREEEEK E-----EVAE AEG-------
macfPPARD  ---------- -MEQPQEE-- APEVREEEEK E-----EVAE AEG-------
macmPPARD  ---------- -MEQPQEE-- APEVREEEEK E-----EVAE AEG-------
btPPARD    ---------- -MEQPPEE-- APEARPEEEK E-----EAAK AEG-------
ssPPARD    ---------- -MEQPPEE-- APEVREEEKK K-----EVAE AEG-------
clPPARD    ---------- -MEQPPGE-- AAEVREEEEK K-----EVAE AEG-------
omPPARD    ---------- -M-KSSDE-- --EVVQQEQE D-----QQEK GSS-------
olPPARD    -SAGSDSCG- -ETSGFELTD VQELKTRESD DEEGKEEAPP SKSPKRDPKK
drPPARDb   ESASSDCGGT SDLQEPSSVS GEEVEGSETS DITGLEASPG SKGSWGGNQQ
drPPARDa   GDSSPCSSQG EGPDDQRLQD VLCELGEGAG LNLELESEGG VA-------L
xlPPARD    ---------- ---------- ---------- ---------- ----------
salsPPARD  ---------- ---------- ---------- ---------- ----------
Consistency 0000000000 0445335300 3244335644 4000005443 4450000000

           ........110.........120.........130.........140.........150
mmPPARD    -----APELN GGPEHTLPSS SCADLSQNSS PSS-LLDQLQ MGCDGASGGS
rnPPARD    -----APELN GGPEHTLPSS SCTDLSQNSS PSS-LLDQLQ MGCDGASGGS
ggPPARD    -----ASDPS AGPDSSLPSS SYTDLSQSSS P-S-LSDQLQ MGCEETASGA
hsPPARD    -----APELN GGPQHALPSS SYTDLSRSSS PPS-LLDQLQ MGCDGASCGS
macfPPARD  -----APELN GGPQHALPSS SYTDLSRSSS PPS-LLDQLQ MGCDGASCGS
macmPPARD  -----APELN GGPQHALPSS SYTDLSRSSS PPS-LLDQLQ MGCDGASCGS
btPPARD    -----APELN GGPEHSLPSS SYTDLSQSCS PLS-LLDQLQ MGCDGASCGS
ssPPARD    -----GPELN GGPEHSLPSS SCTDLSQSCS PPA-LLDQLQ MGCDGASCGG
clPPARD    -----APELN GGPERSLPSS SYTDLSRSSS PPS-LLDQLQ MGGDGASCGS
omPPARD    -----NSHST SNMDSPALSS SCTDLSQTSS P-S-LSDQLL LGREDGTGAL
olPPARD    REGECQDQEN NQNKQNNNAS SSYTDLSHTS SPS-LSEQLR LGREDNAGSG
drPPARDb   NGDRTPQESP PPNSETPVTA GAFSDPLQVP SLS-LSDQLR LGREDPNKLG
drPPARDa   QGEELSWETS RQEEQSNTSS TSPDRSSAST DLS-TALSEQ LLRGREEVSG
xlPPARD    ---------- ---------- ----MKEEIP PRSPILDEQP STPLEHQETS
salsPPARD  ---------- ---------- ---------- ---------- ----------
Consistency 0000435335 4444444467 6346565556 6280757766 6635445355

           ........160.........170.........180.........190.........200
mmPPARD    LNMECRVCGD KASGFHYGVH ACEGCKGFFR RTIRMKLKYE KCDRICKIQK
rnPPARD    LNMECRVCGD KASGFHYGVH ACEGCKGFFR RTIRMKLKYE KCDRICKIQK
ggPPARD    LNVECRVCGD KASGFHYGVH ACEGCKGFFR RTIRMKLEYE KCERSCKIQK
hsPPARD    LNMECRVCGD KASGFHYGVH ACEGCKGFFR RTIRMKLEYE KCERSCKIQK
macfPPARD  LNMECRVCGD KASGFHYGVH ACEGCKGFFR RTIRMKLEYE KCERSCKIQK
macmPPARD  LNMECRVCGD KASGFHYGVH ACEGCKGFFR RTIRMKLEYE KCERSCKIQK
btPPARD    LNMECRVCGD KASGFHYGVH ACEGCKGFFR RTIRMKLEYE KCERICKIQK
ssPPARD    LSMECRVCGD KASGFHYGVH ACEGCKGFFR RTIRMKLEYE KCERICKIQK
clPPARD    LNMECRVCGD KASGFHYGVH ACEGCKGFFR RTIRMKLEYE KCERICKIQK
omPPARD    INVECRICGD KASGFHYGVH ACEGCKGFFR RTIRMKLDYE RCERSCKIQK
olPPARD    ISVECKVCGD KASGFHYGVH ACEGCKGFFR RTVRMKLEYE RCERSCKIQK
drPPARDb   LNVECRICGD KASGFHYGVH ACEGCKGFFR RTIRMKLEYE RCERACKVQK
drPPARDa   INMECRICGD RASGFHYGVH ACEGCKGFFR RTIRMKLEYE RCERSCKIQK
xlPPARD    QSVDCKICGD RASGFHYGVH ACEGCKGFFR RTIRMRLQYE HCDRNCKIQK
salsPPARD  ---------- ---------- ---------- ---------- -CERRCKIQK
Consistency 6768878888 7888888888 8888888888 8888888788 6*8*5**9**
```

```
           ..........210.......220.......230.......240.......250
mmPPARD    KNRNKCQYCR FQKCLALGMS HNAIRFGRMP EAEKRKLVAG LTASE-GCQH
rnPPARD    KNRNKCQYCR FQKCLALGMS HNAIRFGRMP EAEKRKLVAG LTASE-GCQQ
ggPPARD    KNRNKCQYCR FQKCLSLGMS HNAIRFGRMP EAEKRKLVAG LTASE-ISCQ
hsPPARD    KNRNKCQYCR FQKCLALGMS HNAIRFGRMP EAEKRKLVAG LTANE-GSQY
macfPPARD  KNRNKCQYCR FQKCLALGMS HNAIRFGRMP EAEKRKLVAG LTANE-GSQY
macmPPARD  KNRNKCQYCR FQKCLALGMS HNAIRFGRMP EAEKRKLVAG LTANE-GSQY
btPPARD    KNRNKCQYCR FQKCLALGMS HNAIRFGRMP EAEKRKLVAG LTANE-GSQH
ssPPARD    KNRNKCQYCR FQKCLALGMS HNAIRFGRMP EAEKRKLVAG LTANE-GSQH
clPPARD    KNRNKCQYCR FQKCVALGMS HNAIRFGRMP EAEKRKLVAG LTANE-GTQH
omPPARD    KSRNKCQYCR FQKCLLLGMS HDAIRYGRMP EAEKRKLVAG LLAGEKAPTT
olPPARD    KNRNKCQYCR FQKCLSLGMS HDAIRYGRMP EAERKKLVAG LLAEE-MNLS
drPPARDb   KSRNKCQYCR FQKCLALGMS HDAIRYGRMP EAEKKKLVAG LLAGE--NPQ
drPPARDa   KSRNKCQFCR FQKCLMLGMS HDAIRYGRMP EAEKRKLVAG LLAGE-RGSQ
xlPPARD    KNRNKCQYCR FNKCLSLGMS HNAIRFGRMP ESEKRKLVQA PVSDS--AAP
salsPPARD  KSRNKCQYCR FQKCLLLGMS HDAIRYGRMP EAEKRKLVAG LLAGDTAPTT
Consistency *8*****9** *9**96**** *7***8**** *9*99***99 8695803454
```

```
           ..........260.......270.......280.......290.......300
mmPPARD    NPQLADLKAF SKHIYNAYLK NFNMTKKKAR SILTGKSSHN A-----PFVI
rnPPARD    NPQLADLKAF SKHIYNAYLK NFNMTKKKAR SILTGKSSHN A-----PFII
ggPPARD    NPQVADLKAF SKHIYNAYLK NFNMTKKKAR GILTGKASST P----QPFVI
hsPPARD    NPQVADLKAF SKHIYNAYLK NFNMTKKKAR SILTGKASHT A-----PFVI
macfPPARD  NPQVADLKAF SKHIYNAYLK NFNMTKKKAR SILTGKASHT A-----PFVI
macmPPARD  NPQVADLKAF SKHIYNAYLK NFNMTKKKAR SILTGKASHT A-----PFVI
btPPARD    NPQVADLRAF SKHIYSAYLK NFNMTKKKAR GILTGKASHT A-----PFVI
ssPPARD    NPQVADLKAF SKHLYSAYLK NFNMTKKKAR AILTGKASHT A-----PFVI
clPPARD    NPQVADLKAF SKHIYNAYLK NFNMTKKKAR GILTGKASHT A-----PFVI
omPPARD    NPNSSDLKSL AKRVNNAYLK NLNMTKKKAR SILTGKTSSS PVAYPYPFVI
olPPARD    KPGGSDLKTL AKQVNTAYQK NLSMTKKKAR SILTGKTSST S-----PFVI
drPPARDb   SSSGADLKTL AKHVNTAYLR NLNMTKKKAR SILTGKTSCT A-----PFVI
drPPARDa   NSNESDLKTL AKRVNNAYLK NLNMTKKKAR NILTGKTSAS T-----PFVI
xlPPARD    DSPVSDLDVL SQLIHSSYMN TFTMTKKRAR DILTGRNSIS ------PFVI
salsPPARD  NPNGSDLKSL AKRVNNAYLK NLNMTKKKAR SILTGKNSSS PVAYPYPFVI
Consistency 87648**877 8969579*88 978****9** 6****96*57 500000**9*
```

```
           ..........310.......320.......330.......340.......350
mmPPARD    HDIETLWQAE KGLVWKQLVN GLPPYNEISV HVFYRCQSTT VETVRELTEF
rnPPARD    HDIETLWQAE KGLVWKQLVN GPPPYNEISV HVFYRCQSTT VETVRELTEF
ggPPARD    HDMDTLWQAE KGLVWKQLVN GIPPYKEIGV HVFYRCQCTT VETVRELTEF
hsPPARD    HDIETLWQAE KGLVWKQLVN GLPPYKEISV HVFYRCQCTT VETVRELTEF
macfPPARD  HDIETLWQAE KGLVWKQLVN GLPPYKEISV HVFYRCQCTT VETVRELIEF
macmPPARD  HDIETLWQAE KGLVWKQLVN GLPPYKEISV HVFYRCQCTT VETVRELIEF
btPPARD    HDIETLWQAE KGLVWKQLVN SLPPYKEISV HVFYRCQCTT VETVRELTEF
ssPPARD    HDIETLWQAE KGLVWKQLVN GLPPYKEISV HVFYRCQCTT VETVRELTEF
clPPARD    HDIETLWQAE KGLVWKQLVN GLPPYKEISV HVFYRCQCTT VETVRELTEF
omPPARD    HDMDSLCQAE NGLVWKQLIN GTTPNKEIGV HVFYRCQCTT VETVRELTEF
olPPARD    YDVETLWKAE SGLVWSQLVP GAPLTKEIGV HVFYRCQCTT VETVRELTEF
drPPARDb   HDMDSLWQAE NGLVWNQLNG A-PLNKEIGV HVFYRCQCTT VETVRELTEF
drPPARDa   HDMDSLVQAE NGLVWNQVIH GAPPNKEIGV HVFYRCQCTT VETVRELTEF
xlPPARD    HDMDTLWQAE QGTVWEQLPT QNLTGTEIGV HVFYRCQCTS VETVRALTDF
salsPPARD  HDMDSLHQAE NGLVWNQLIN GTPPNKEIGV HVFYRCQCTT VETVRELTEF
Consistency 9*788*69** 6*9**7*976 747757**7* *******8*9 *****9*89*
```

```
           ..........360.......370.......380.......390.......400
mmPPARD    AKNIPNFSSL FLNDQVTLLK YGVHEAIFAM LASIVNKDGL LVANGSGFVT
rnPPARD    AKNIPNFSSL FLNDQVTLLK YGVHEAIFAM LASIVNKDGL LVANGSGFVT
ggPPARD    AKSIPSFIGL YLNDQVTLLK YGVHEAIFAM LASIMNKDGL LVANGNGFVT
hsPPARD    AKSIPSFSSL FLNDQVTLLK YGVHEAIFAM LASIVNKDGL LVANGSGFVT
macfPPARD  AKSIPSFSSL FLNDQVTLLK YGVHEAIFAM LASIVNKDGL LVANGSGFVT
macmPPARD  AKSIPSFSSL FLNDQVTLLK YGVHEAIFAM LASIVNKDGL LVANGSGFVT
btPPARD    AKSIPSFGDL FLNDQVTLLK YGVHEAIFAM LASIVNKDGL LVANGTGFVT
ssPPARD    AKSIPSFDHL FLNDQVTLLK YGVHEAIFAM LASIVNKDGL LVANGTGFVT
clPPARD    AKSIPSFSNL FLNDQVTLLK YGVHEAIFAM LASIVNKDGL LVANGTGFVT
omPPARD    AKSIPGFVDL FLNDQVTLLK YGVHEAIFAM LPSLMNKDGL LVANGKGFVT
olPPARD    AKCIPGFVDL YLNDQVTLLK YGVHGAIFAM LPSLMNKDGL LVANGKGFVT
drPPARDb   AKNIPGFVDL FLNDQVTLLK YGVHEAIFAM LPSLMNKDGL LVANGKGFVT
drPPARDa   SKSIPGFIDL FLNDQVTLLK YGVHEAIFAM LPSLMNKDGL LVANGRGFVT
xlPPARD    AKRIPGFGTL YLNDQVTLLK YGVHEAIFCM LASLMNKDGL LVAGGRGFVT
salsPPARD  AKSIPGFVDL FLNDQVTLLK YGVHEAIFAM LPSLMNKDGL LVANGKGFVT
Consistency 9*6**6*45* 8********* ****8***9* *6*87***** ***9*5****
```

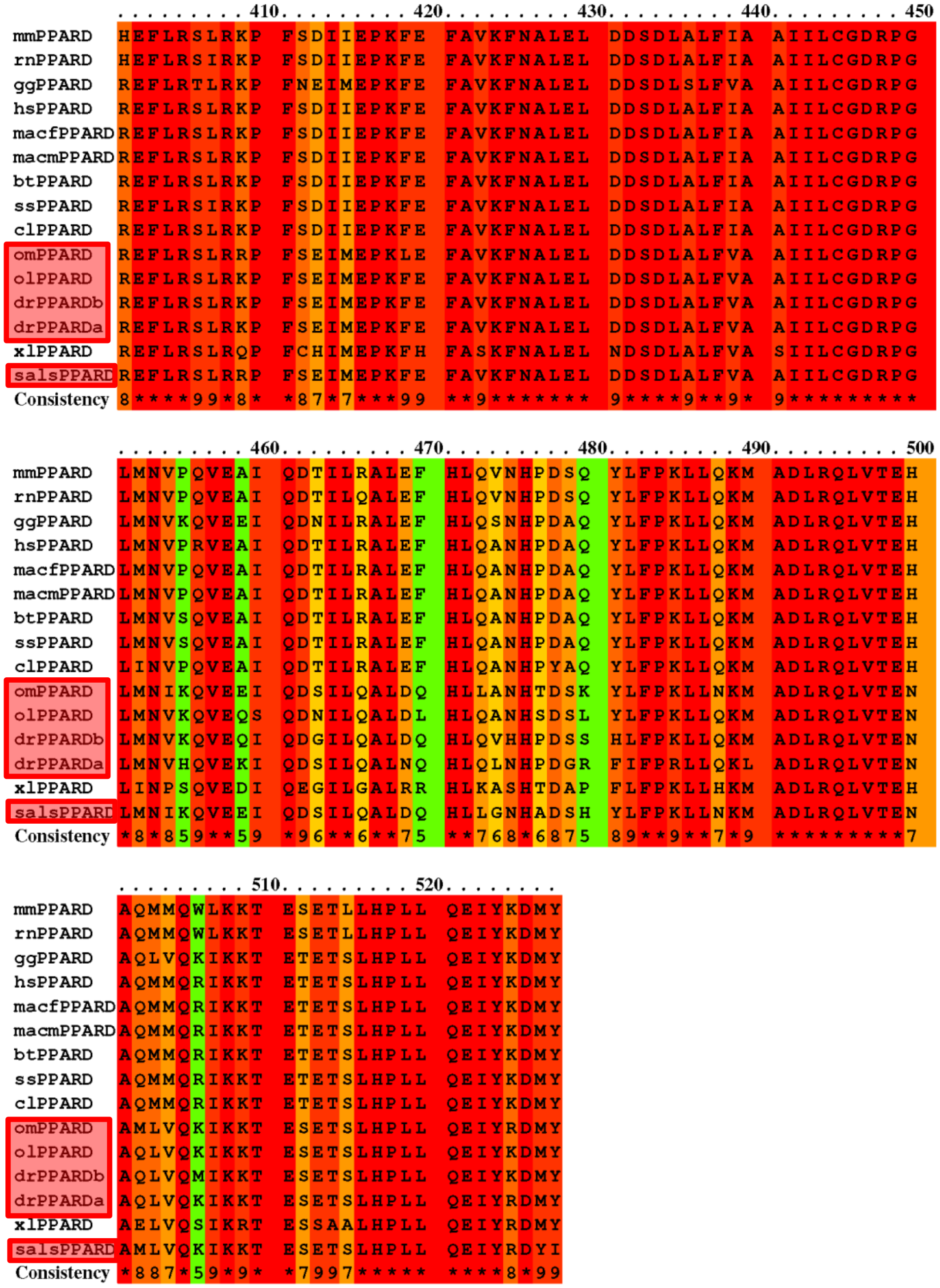

410 420 430 440 450
mmPPARD HEFLRSLRKP FSDIIEPKFE FAVKFNALEL DDSDLALFIA AIILCGDRPG
rnPPARD HEFLRSIRKP FSDIIEPKFE FAVKFNALEL DDSDLALFIA AIILCGDRPG
ggPPARD REFLRTLRKP FNEIMEPKFE FAVKFNALEL DDSDLSLFVA AIILCGDRPG
hsPPARD REFLRSLRKP FSDIIEPKFE FAVKFNALEL DDSDLALFIA AIILCGDRPG
macfPPARD REFLRSLRKP FSDIIEPKFE FAVKFNALEL DDSDLALFIA AIILCGDRPG
macmPPARD REFLRSLRKP FSDIIEPKFE FAVKFNALEL DDSDLALFIA AIILCGDRPG
btPPARD REFLRSLRKP FSDIIEPKFE FAVKFNALEL DDSDLALFIA AIILCGDRPG
ssPPARD REFLRSIRKP FSDIIEPKFE FAVKFNALEL DDSDLALFIA AIILCGDRPG
clPPARD REFLRSLRKP FSDIIEPKFE FAVKFNALEL DDSDLALFIA AIILCGDRPG
omPPARD REFLRSLRRP FSEIMEPKLE FAVKFNALEL DDSDLALFVA AIILCGDRPG
olPPARD REFLRSLRKP FSEIMEPKFE FAVKFNALEL DDSDLALFVA AIILCGDRPG
drPPARDb REFLRSLRKP FSEIMEPKFE FAVKFNALEL DDSDLALFVA AIILCGDRPG
drPPARDa REFLRSLRKP FSEIMEPKFE FAVKFNALEL DDSDLALFVA AIILCGDRPG
xlPPARD REFLRSLRQP FCHIMEPKFH FASKFNALEL NDSDLALFVA SIILCGDRPG
salsPPARD REFLRSLRRP FSEIMEPKFE FAVKFNALEL DDSDLALFVA AIILCGDRPG
Consistency 8****99*8* *87*7***99 **9******* 9****9**9* 9*********
460 470 480 490 500
mmPPARD LMNVPQVEAI QDTILRALEF HLQVNHPDSQ YLFPKLLQKM ADLRQLVTEH
rnPPARD LMNVPQVEAI QDTILQALEF HLQVNHPDSQ YLFPKLLQKM ADLRQLVTEH
ggPPARD LMNVKQVEEI QDNILRALEF HLQSNHPDAQ YLFPKLLQKM ADLRQLVTEH
hsPPARD LMNVPRVEAI QDTILRALEF HLQANHPDAQ YLFPKLLQKM ADLRQLVTEH
macfPPARD LMNVPQVEAI QDTILRALEF HLQANHPDAQ YLFPKLLQKM ADLRQLVTEH
macmPPARD LMNVPQVEAI QDTILRALEF HLQANHPDAQ YLFPKLLQKM ADLRQLVTEH
btPPARD LMNVSQVEAI QDTILRALEF HLQANHPDAQ YLFPKLLQKM ADLRQLVTEH
ssPPARD LMNVSQVEAI QDTILRALEF HLQANHPDAQ YLFPKLLQKM ADLRQLVTEH
clPPARD LINVPQVEAI QDTILRALEF HLQANHPYAQ YLFPKLLQKM ADLRQLVTEH
omPPARD LMNIKQVEEI QDSILQALDQ HLLANHTDSK YLFPKLLNKM ADLRQLVTEN
olPPARD LMNVKQVEQS QDNILQALDL HLQANHSDSL YLFPKLLQKM ADLRQLVTEN
drPPARDb LMNVKQVEQI QDGILQALDQ HLQVHHPDSS HLFPKLLQKM ADLRQLVTEN
drPPARDa LMNVHQVEKI QDSILQALNQ HLQLNHPDGR FIFPRLLQKL ADLRQLVTEN
xlPPARD LINPSQVEDI QEGILGALRR HLKASHTDAP FLFPKLLHKM ADLRQLVTEH
salsPPARD LMNIKQVEEI QDSILQALDQ HLLGNHADSH YLFPKLLNKM ADLRQLVTEN
Consistency *8*859**59 *96**6**75 **768*6875 89**9**7*9 *********7
510 520
mmPPARD AQMMQWLKKT ESETLLHPLL QEIYKDMY
rnPPARD AQMMQWLKKT ESETLLHPLL QEIYKDMY
ggPPARD AQLVQKIKKT ETETSLHPLL QEIYKDMY
hsPPARD AQMMQRIKKT ETETSLHPLL QEIYKDMY
macfPPARD AQMMQRIKKT ETETSLHPLL QEIYKDMY
macmPPARD AQMMQRIKKT ETETSLHPLL QEIYKDMY
btPPARD AQMMQRIKKT ETETSLHPLL QEIYKDMY
ssPPARD AQMMQRIKKT ETETSLHPLL QEIYKDMY
clPPARD AQMMQRIKKT ETETSLHPLL QEIYKDMY
omPPARD AMLVQKIKKT ESETSLHPLL QEIYRDMY
olPPARD AQLVQKIKKT ESETSLHPLL QEIYKDMY
drPPARDb AQLVQMIKKT ESETSLHPLL QEIYKDMY
drPPARDa AQLVQKIKKT ESETSLHPLL QEIYRDMY
xlPPARD AELVQSIKRT ESSAALHPLL QEIYRDMY
salsPPARD AMLVQKIKKT ESETSLHPLL QEIYRDYI
Consistency *887*59*9* *7997***** ****8*99

Supplemental data 2C: Protein alignment PPARG

Unconserved 0 1 2 3 4 5 6 7 8 9 10 Conserved

```
            .........10 .........20 .........30 .........40 .........50
mmPPARGi1   ---------- ---------- ---------- MVDTEMPF-W -PTNFGISSV
rnPPARGi2   ---------- ---------- ---------- MVDTEMPF-W -PTNFGISSV
macfPPARG   ---------- ---------- ---------- MVDTEMPF-W -PTNFGISSV
ggPPARG     ---------- ---------- ---------- MVDTEMPF-W -PVNFGISPV
ocPPARG     ---------- ---------- ---------- MVDTEMPF-W -PTNFGIGSV
mmPARGi2    MGETLGDSPV DPEHGAFADA LPMSTSQEIT MVDTEMPF-W -PTNFGISSV
rnPPARGi1   MGETLGDPPV DPEHGAFADA LPMSTSQEIT MVDTEMPF-W -PTNFGISSV
btPPARGi2   MGETLGDALI DPESEPFAVT VSARTSQEIT MVDTEMPF-W -PTNFGISSV
clPPARG     MGETLGDSLI DPESDSFADT LSASTSQETT MVDTEMPF-W -PTNFGISSV
ssPPARG     MGETLGDSLI DPESDAF-DT LSANISQEVT MVDTEMPF-W -PTNFGISSV
hsPPARGi2   MGETLGDSPI DPESDSFTDT LSANISQEMT MVDTEMPF-W -PTNFGISSV
macmPPARG   MGETLGDSPI DPESDSFTDT LSANISQEIT MVDTEMPF-W -PTNFGISSV
drPPARG     ---------- -MLHRRFS-- -VQKASLSAD MVDTQTFG-W -PVGFGLSAL
hsPPARGi1   ---------- ---------- --------MT MVDTEMPF-W -PTNFGISSV
btPPARGi1   ---------- ---------- ---------- MVDTEMPF-W -PTNFGISSV
xlPPARG     ---------- ---------- ---------- MVDTEMPF-W SNLNFGMNSM
olPPARG     ---------- ---------- ---------- MVDTQQLLAW P-VGFSLSTV
salsPPARG   ---------- ---------- ---------- MVDTRRAA-W SLLSFGLGTL
omPPARG     ---------- ---------- ---------- MVDTRRAA-W SLLSFGLGTL
Consistency 1111111001 1111001000 1010011111 ****87670* 0678*98878

            .........60 .........70 .........80 .........90 ........100
mmPPARGi1   DLSVMEDHSH SFDIKPFTTV DFSSISAPHY E--------- ----------
rnPPARGi2   DLSVMDDHSH SFDIKPFTTV DFSSISAPHY E--------- ----------
macfPPARG   DLSVMDDHSH SFDIKPFTTV DFSSISAPHY E--------- ----------
ggPPARG     DLSAMDDHMH SFDIKPFTTV DFSSISSPHY E--------- ----------
ocPPARG     DLSVMDDHSH SFDIKPFTTV DFSSISAPHY E--------- ----------
mmPARGi2    DLSVMEDHSH SFDIKPFTTV DFSSISAPHY E--------- ----------
rnPPARGi1   DLSVMDDHSH SFDIKPFTTV DFSSISAPHY E--------- ----------
btPPARGi2   DLSMMDDHSH AFDIKPFTTV DFSSISTPHY E--------- ----------
clPPARG     DLSVMDDHSH SFDIKPFTTV DFSSISTPHY E--------- ----------
ssPPARG     DLSVMDDHSH SFDIKPFTTV DFSSISTPHY E--------- ----------
hsPPARGi2   DLSVMEDHSH SFDIKPFTTV DFSSISTPHY E--------- ----------
macmPPARG   DLSVMDDHSH SFDIKPFTTV DFSSISAPHY E--------- ----------
drPPARG     ELEELEDDTH SLDIKPFSTL DYSSISGIDY E--------- ----------
hsPPARGi1   DLSVMEDHSH SFDIKPFTTV DFSSISTPHY E--------- ----------
btPPARGi1   DLSMMDDHSH AFDIKPFTTV DFSSISTPHY E--------- ----------
xlPPARG     DMSALEDHCQ PYDIKPFTTV DFSSIN-SHY D--------- ----------
olPPARG     DLPEFNDSSH CLDMKHLSTS DYASISSSSI P--------- ----------
salsPPARG   DLVEMDNKMN SFDMKTLSTL DYPYLPSLEY SHNS------ ----------
omPPARG     DLAEMDNKMN SFDMETLSTL DYPYLPSLEY SHNSPHHHSP DRSHSFNHSP
Consistency 9976879778 78*89788*8 *888985679 7000000000 0000000000

            ........110 ........120 ........130 ........140 ........150
mmPPARGi1   ---------- DIP-----FT RADP------ ---------- --MVADYKYD
rnPPARGi2   ---------- DIP-----FT RADP------ ---------- --MVADYKYD
macfPPARG   ---------- DIP-----FT RTDP------ ---------- --MVADYKYD
ggPPARG     ---------- DIP-----LG RADQ------ ---------- --TSIDYKYD
ocPPARG     ---------- DLP-----FA RADP------ ---------- --MVADYKYD
mmPARGi2    ---------- DIP-----FT RADP------ ---------- --MVADYKYD
rnPPARGi1   ---------- DIP-----FT RADP------ ---------- --MVADYKYD
btPPARGi2   ---------- DIP-----FP RADP------ ---------- --MVADYKYD
clPPARG     ---------- DIP-----FS RADP------ ---------- --MVADYKYD
ssPPARG     ---------- DIP-----FP RADP------ ---------- --MVADYKYD
hsPPARGi2   ---------- DIP-----FT RTDP------ ---------- --VVADYKYD
macmPPARG   ---------- DIP-----FT RTDP------ ---------- --MVADYKYD
drPPARG     ---------- NNP------T QNDPT----- ---------P HMMDLTHMYS
hsPPARGi1   ---------- DIP-----FT RTDP------ ---------- --VVADYKYD
btPPARGi1   ---------- DIP-----FP RADP------ ---------- --MVADYKYD
xlPPARG     ---------- DILDEKTFLC RNDQ------ ---------- --SPIDYKYD
olPPARG     ---------- --------SS LSPP------ ---------- LVSSMSSTGM
salsPPARG   ---PHHHHSP DRSHSCNHSP DRSHS----- -----FNHSP DRSHSFNHSP
omPPARG     DRSQSFNHSP DRNHSINHSP DRSYSYNHRS DRSHSFNHSP DRSHSFNHSP
Consistency 0000000000 8660000065 7587000000 0000000000 0065777777
```

```
          .........160.........170.........180.........190.........200
mmPPARGi1 LKLQEY---- ---------- ---------- ---QSAIKVE PASPPYYSEK
rnPPARGi2 LKLQEY---- ---------- ---------- ---QSAIKVE PASPPYYSEK
macfPPARG LKLQEY---- ---------- ---------- ---QSAIKVE PASPPYYSEK
ggPPARG   IKLQDC---- ---------- ---------- ---QSAIKME PPSPPYFSEK
ocPPARG   LKLQEY---- ---------- ---------- ---QSAIKVE PASPPYYSEK
mmPARGi2  LKLQEY---- ---------- ---------- ---QSAIKVE PASPPYYSEK
rnPPARGi1 LKLQEY---- ---------- ---------- ---QSAIKVE PASPPYYSEK
btPPARGi2 LKLQEY---- ---------- ---------- ---QSAIKVE PVSPPYYSEK
clPPARG   LKLQEY---- ---------- ---------- ---QSAIKVE PASPPYYSEK
ssPPARG   LKLQDY---- ---------- ---------- ---QSAIKVE PVSPPYYSEK
hsPPARGi2 LKLQEY---- ---------- ---------- ---QSAIKVE PASPPYYSEK
macmPPARG LKLQEY---- ---------- ---------- ---QSAIKVE PASPPYYSEK
drPPARG   YRTQENYRTH EPIYRPEHSS YSPEENTYRA QQIQNSIKLE PESPPQFAEN
hsPPARGi1 LKLQEY---- ---------- ---------- ---QSAIKVE PASPPYYSEK
btPPARGi1 LKLQEY---- ---------- ---------- ---QSAIKVE PVSPPYYSEK
xlPPARG   LKLQEC---- ---------- ---------- ---QSSIKLE PPSPPYFSDK
olPPARG   AYDPSSPQSE DHLTNMDY-- -TNLHSYRTE LDTHSSVKLE PDSPPQYSDS
salsPPARG DRNHSFNHSP DRSHSFNHSP DRSHSYNDTY SVYQGSVNDK PLSPSQSSDC
omPPARG   DRSQSFNHSP DRNHSINHSP DRSHSYNHRS DRSHSFNHSP DRSHSFNHSP
Consistency 6868760000 0000000000 0000000000 0008878878 95*9877887

          .........210.........220.........230.........240.........250
mmPPARGi1 TQLYNRP--- ---------- ---------- -HEEPSNSLM AIECRVCGDK
rnPPARGi2 TQLYNRP--- ---------- ---------- -HEEPSNSLM AIECRVCGDK
macfPPARG TQLYNKP--- ---------- ---------- -HEEPSNSLM AIECRVCGDK
ggPPARG   VQLYNKP--- ---------- ---------- -HEESSNSLM AIECRVCGDK
ocPPARG   TQLYNKT--- ---------- ---------- -HEEPSNSLM AIECRVCGDK
mmPARGi2  TQLYNRP--- ---------- ---------- -HEEPSNSLM AIECRVCGDK
rnPPARGi1 TQLYNRP--- ---------- ---------- -HEEPSNSLM AIECRVCGDK
btPPARGi2 TQLYSKP--- ---------- ---------- -HEEPSNSLM AIECRVCGDK
clPPARG   TQLYNKP--- ---------- ---------- -HEEPSNSLM AIECRVCGDK
ssPPARG   TQLYNKP--- ---------- ---------- -HEEPSNSLM AIECRVCGDK
hsPPARGi2 TQLYNKP--- ---------- ---------- -HEEPSNSLM AIECRVCGDK
macmPPARG TQLYNKP--- ---------- ---------- -HEEPSNSLM AIECRVCGDK
drPPARG   SVSFSKT--- ---------- ---------- -PEDPSSSSL NIECRVCGDK
hsPPARGi1 TQLYNKP--- ---------- ---------- -HEEPSNSLM AIECRVCGDK
btPPARGi1 TQLYSKP--- ---------- ---------- -HEEPSNSLM AIECRVCGDK
xlPPARG   PQC-SKA--- ---------- ---------- -FEDTPNSFI AIECRVCGDK
olPPARG   PVFSKLP--- ---------- ---------- --EDTPSSSL NIECRVCGDK
salsPPARG SIVSLSRPRP HSNP------ ---------- PTYTDASSLL NIDCRVCGDK
omPPARG   DRSHSFNHRS DRSHSFNHSP DKSHSYNYTY SGNQNASSPL NIDCRVCGDK
Consistency 6776766000 0000000000 0000000000 0688678*78 7*9*******

          .........260.........270.........280.........290.........300
mmPPARGi1 ASGFHYGVHA CEGCKGFFRR TIRLKLIYDR CDLNCRIHKK SRNKCQYCRF
rnPPARGi2 ASGFHYGVHA CEGCKGFFRR TIRLKLIYDR CDLNCRIHKK SRNKCQYCRF
macfPPARG ASGFHYGVHA CEGCKGFFRR TIRLKLIYDR CDLNCRIHKK SRNKCQYCRF
ggPPARG   ASGFHYGVHA CEGCKGFFRR TIRLKLIYDR CDLNCRIHKK SRNKCQYCRF
ocPPARG   ASGFHYGVHA CEGCKGFFRR PIRLKLIYDR CDLNCRIHKK SRNKCQYCRF
mmPARGi2  ASGFHYGVHA CEGCKGFFRR TIRLKLIYDR CDLNCRIHKK SRNKCQYCRF
rnPPARGi1 ASGFHYGVHA CEGCKGFFRR TIRLKLIYDR CDLNCRIHKK SRNKCQYCRF
btPPARGi2 ASGFHYGVHA CEGCKGFFRR TIRLKLIYDR CDLNCRIHKK SRNKCQYCRF
clPPARG   ASGFHYGVHA CEGCKGFFRR TIRLKLIYDR CDLNCRIHKK SRNKCQYCRF
ssPPARG   ASGFHYGVHA CEGCKGFFRR TIRLKLIYDR CDLNCRIHKK SRNKCQYCRF
hsPPARGi2 ASGFHYGVHA CEGCKGFFRR TIRLKLIYDR CDLNCRIHKK SRNKCQYCRF
macmPPARG ASGFHYGVHA CEGCKGFFRR TIRLKLIYDR CDLNCRIHKK SRNKCQYCRF
drPPARG   ASGFHYGVHA CEGCKGFFRR TIRLKLVYDH CDLHCRIHKK SRNKCQYCRF
hsPPARGi1 ASGFHYGVHA CEGCKGFFRR TIRLKLIYDR CDLNCRIHKK SRNKCQYCRF
btPPARGi1 ASGFHYGVHA CEGCKGFFRR TIRLKLIYDR CDLNCRIHKK SRNKCQYCRF
xlPPARG   ASGFHYGVHA CEGCKGFFRR TIRLKLIYER CDLNCRIHKK SRNKCQFCRF
olPPARG   ASGFHYGVHA CEGCKGFFRR TIRLKLVYDH CDLHCRIHKK SRNKCQYCRF
salsPPARG ASGFHYGVHV CEGCKGFFRR TVRLKLVYDH CDLHCRIHKK SRNKCQYCRF
omPPARG   ASGFHYGVHA CEGCKGFFRR TVRLKLVYDH CELHCRIHKK SRNKCQYCRF
Consistency *********9 ********** 99****9*97 *9*8****** ******9***

          .........310.........320.........330.........340.........350
mmPPARGi1 QKCLAVGMSH NAIRFGRMPQ AEKEKLLAEI SSDIDQLNPE SADLRALAKH
rnPPARGi2 QKCLAVGMSH NAIRFGRMPQ AEKEKLLAEI SSDIDQLNPE SADLRALAKH
macfPPARG QKCLAVGMSH NAIRFGRMPQ AEKEKLLAEI SSDIDQLNPE SADLRALAKH
ggPPARG   QKCLAVGMSH NAIRFGRMPQ AEKEKLLAEI SSDIDQLNPE SADLRALAKH
ocPPARG   QKCLAVGMSH NAIRFGRMPG AEKEKLLAEI SSDIDQLNPE SADLRALAKH
mmPARGi2  QKCLAVGMSH NAIRFGRMPQ AEKEKLLAEI SSDIDQLNPE SADLRALAKH
rnPPARGi1 QKCLAVGMSH NAIRFGRMPQ AEKEKLLAEI SSDIDQLNPE SADLRALAKH
btPPARGi2 QKCLAVGMSH NAIRFGRMPQ AEKEKLLAEI SSDIDQLNPE SADLRALAKH
clPPARG   QKCLAVGMSH NAIRFGRMPQ AEKEKLLAEI SSDIDQLNPE SADLRALAKH
ssPPARG   QKCLAVGMSH NAIRFGRMPQ AEKEKLLAEI SSDIDQLNPE SADLRALAKH
hsPPARGi2 QKCLAVGMSH NAIRFGRMPQ AEKEKLLAEI SSDIDQLNPE SADLRALAKH
macmPPARG QKCLAVGMSH NAIRFGRMPQ AEKEKLLAEI SSDIDQLNPE SADLRALAKH
drPPARG   QKCLMVGMSH NAIRFGRMPQ AEKEKLLAEF SSDVNHMHPE SADLRALARH
hsPPARGi1 QKCLAVGMSH NAIRFGRMPQ AEKEKLLAEI SSDIDQLNPE SADLRALAKH
btPPARGi1 QKCLAVGMSH NAIRFGRMPQ AEKEKLLAEI SSDIDQLNPE SADLRALAKH
xlPPARG   QKCLAVGMSH NAIRFGRMPQ AEKEKLLAEI SSDIDQLNPE SADQRVLAKH
olPPARG   QKCLNVGMSH NAIRFGRMPQ AEKEKLLAEF SSDMEHMHPE AADLRALARH
salsPPARG QKCLLVGMSH DAIRFGRMPQ VEREKLLQAE FMDVEPRNPE SADLRALSRQ
omPPARG   QKCLLVGMSH DAIRFGRMPQ VEREKLQAEF MD-VDPRNPE SAELRALSRQ
Consistency ****7***** 9********9 9*9***9997 88898778** 9*99*9*988
```

```
          .........360.........370.........380.........390.........400
mmPPARGi1 LYDSYIKSFP LTKAKARAIL TGKTTDKSPF VIYDMNSLMM GEDKIKFKHI
rnPPARGi2 LYDSYIKSFP LTKAKARAIL TGKTTDKSPF VIYDMNSLMM GEDKIKFKHI
macfPPARG LYDSYIKSFP LTKAKARAIL TGKTTDKSPF VIYDMNSLMM GEDKIKFKHI
ggPPARG   LYDSYIKSFP LTKAKARAIL TGKTTDKSPF VIYDMNSLRM GEDQIKCKHA
ocPPARG   LYDSYIKSFP LTKAKARAIL TGKTTDKSPF VIYDMNSLMM GEDKIKFKHI
mmPARGi2  LYDSYIKSFP LTKAKARAIL TGKTTDKSPF VIYDMNSLMM GEDKIKFKHI
rnPPARGi1 LYDSYIKSFP LTKAKARAIL TGKTTDKSPF VIYDMNSLMM GEDKIKFKHI
btPPARGi2 LYDSYIKSFP LTKAKARAIL TGKTTDKSPF VIYDMNSLMM GEDKIKFKHI
clPPARG   LYDSYIKSFP LTKAKARAIL TGKTTDKSPF VIYDMNSLMM GEDKIKFKHI
ssPPARG   LYDSYIKSFP LTKAKARAIL TGKTTDKSPF VIYDMNSLMM GEDKIKFKHI
hsPPARGi2 LYDSYIKSFP LTKAKARAIL TGKTTDKSPF VIYDMNSLMM GEDKIKFKHI
macmPPARG LYDSYIKSFP LTKAKARAIL TGKTTDKSPF VIYDMNSLMM GEDKIKFKHI
drPPARG   LYESYLKYFP LTKAKARAIL SGKTSDNAPF VIHDMKSLVE GEQMINCRYM
hsPPARGi1 LYDSYIKSFP LTKAKARAIL TGKTTDKSPF VIYDMNSLMM GEDKIKFKHI
btPPARGi1 LYDSYIKSFP LTKAKARAIL TGKTTDKSPF VIYDMNSLMM GEDKIKFKHI
xlPPARG   LYDSYVKSFP LTKAKARAIL TGRATDKTPV VIHDMNSLMM GEDQIKGQCV
olPPARG   LYEAYLKYFP LTKAKARAIL SGKSGDNVPF VIHDIKSLME GKQLINNGQT
salsPPARG LCLSYHRHFP LTKSKAKAIL SGKTHGNSPF VIHDMKSLTA GQYFINCRQL
omPPARG   LCLSYHRHFP LTKSKAKAIL SGKTHGNSPF VIHDMKSLTA GQYFINCRQL
Consistency *879*797** ***9**9*** 8*997878*9 **8*97**77 *876*75777

          .........410.........420.........430.........440.........450
mmPPARGi1 ---------- ---------- ---------- ---TPLQEQS KEVAIRIFQG
rnPPARGi2 ---------- ---------- ---------- ---TPLQEQS KEVAIRIFQG
macfPPARG ---------- ---------- ---------- ---TPLQEQS KEVAIRIFQG
ggPPARG   ---------- ---------- ---------- ---SPLQEQN KEVAIRIFQR
ocPPARG   ---------- ---------- ---------- ---TPLQEQS KEVAIRIFQG
mmPARGi2  ---------- ---------- ---------- ---TPLQEQS KEVAIRIFQG
rnPPARGi1 ---------- ---------- ---------- ---TPLQEQS KEVAIRIFQG
btPPARGi2 ---------- ---------- ---------- ---SPLQEPS KEVAIRIFQG
clPPARG   ---------- ---------- ---------- ---TPLQEQS KEVAIRIFQG
ssPPARG   ---------- ---------- ---------- ---TPLQEQS KEVAIRIFQG
hsPPARGi2 ---------- ---------- ---------- ---TPLQEQS KEVAIRIFQG
macmPPARG ---------- ---------- ---------- ---TPLQEQS KEVAIRIFQG
drPPARG   PLHEHR---- ---------- ---------- ---RSDLGIM HEVELRFFHS
hsPPARGi1 ---------- ---------- ---------- ---TPLQEQS KEVAIRIFQG
btPPARGi1 ---------- ---------- ---------- ---SPLQEPS KEVAIRIFQG
xlPPARG   ---------- ---------- ---------- -----TPEQN KEVAIRIFQR
olPPARG   SFQEQQIATA GHGVSGIAGA LSGPDCNVFG MASNIGSEPP DGVELRFFYS
salsPPARG PVLERQRSV- ---------- ---------- ---LPPEEPA EELELSVFRR
omPPARG   PVLERQRSV- ---------- ---------- ---LPPEEPA GELELSVFRR
Consistency 0000000000 0000000000 0000000000 0005757967 7997988*76

          .........460.........470.........480.........490.........500
mmPPARGi1 CQFRSVEAVQ EITEYAKNIP GFINLDLNDQ VTLLKYGVHE IIYTMLASLM
rnPPARGi2 CQFRSVEAVQ EITEYAKNIP GFINLDLNDQ VTLLKYGVHE IIYTMLASLM
macfPPARG CQFRSVEAVQ EITEYAKSIP GFVNLDLNDQ VTLLKYGVHE IIYTMLASLM
ggPPARG   CQFRSVEAVQ EITEFAKNIP GFVNLDLNDQ VTLLKYGVHE IIYTLLASLM
ocPPARG   CQFRSVEAVQ EITEYAKNIP GFVSLDLNDQ VTLLKYGVHE IIYTMLASLM
mmPARGi2  CQFRSVEAVQ EITEYAKNIP GFINLDLNDQ VTLLKYGVHE IIYTMLASLM
rnPPARGi1 CQFRSVEAVQ EITEYAKNIP GFINLDLNDQ VTLLKYGVHE IIYTMLASLM
btPPARGi2 CQFRSVEAVQ EITEYAKNIP GFVNLDLNDQ VTLLKYGVHE IIYTMLASLM
clPPARG   CQFRSVEAVQ EITEYAKSIP GFVNLDLNDQ VTLLKYGVHE IIYTMLASLM
ssPPARG   CQFRSVEAVQ EITEYAKNIP GFVNLDLNDQ VTLLKYGVHE IIYTMLASLM
hsPPARGi2 CQFRSVEAVQ EITEYAKSIP GFVNLDLNDQ VTLLKYGVHE IIYTMLASLM
macmPPARG CQFRSVEAVQ EITEYAKSIP GFVNLDLNDQ VTLLKYGVHE IIYTMLASLM
drPPARG   YQSRSAEAIS EVTEFAKSIP GFINLDLNDQ VTLLKYGVIE VMIIMISPLM
hsPPARGi1 CQFRSVEAVQ EITEYAKSIP GFVNLDLNDQ VTLLKYGVHE IIYTMLASLM
btPPARGi1 CQFRSVEAVQ EITEYAKNIP GFVNLDLNDQ VTLLKYGVHE IIYTMLASLM
xlPPARG   CQSRSAEAVR EITEFAKNIP GFVSLDLNDQ VTLLKYGVHE IIFTMLASLM
olPPARG   CQSRSAEAVR EVTEFAKSIP GFVNLDLNDQ VTLLKYGVIE VLILMMSPLM
salsPPARG IQFRSAEAVQ EVTEFTKSIP GFTELDMNDQ VILLKYGVIE VMTTMLAPLM
omPPARG   IQIRSAEAVQ EVTEFAKSIP GFTELDMNDQ VILLKYGVIE VMTTMLAPLM
Consistency 7*7**7**98 *9**89*7** **88**9*** *8******6* 98689997**
```

```
            .........510.........520.........530.........540.........550
mmPPARGi1   NKDGVLISEG QGFMTREFLK SLRKPFGDFM EPKFEFAVKF NALELDDSDL
rnPPARGi2   NKDGVLISEG QGFMTREFLK SLRKPFGDFM EPKFEFAVKF NALELDDSDL
macfPPARG   NKDGVFISEG QGFMTREFLK SLRKPFGDFM EPKFEFAVKF NALELDDSDL
ggPPARG     NKDGVLISDG QGFMTREFLK SLRKPFCDFM EPKFEFAVKF NALELDDSDL
ocPPARG     NKDGVLISEG QGFMTREFLK SLRKPFGDFM EPKFEFAVKF NALELDDSDL
mmPARGi2    NKDGVLISEG QGFMTREFLK SLRKPFGDFM EPKFEFAVKF NALELDDSDL
rnPPARGi1   NKDGVLISEG QGFMTREFLK SLRKPFGDFM EPKFEFAVKF NALELDDSDL
btPPARGi2   NKDGVLISEG QGFMTREFLK SLRKPFGDFM EPKFEFAVKF NALELDDSDL
clPPARG     NKDGVLISEG QGFMTREFLK SLRKPFGDFM EPKFEFAVKF NALELDDSDL
ssPPARG     NKDGVLISEG QGFMTREFLK SLRKPFGDFM EPKFEFAVKF NALELDDSDL
hsPPARGi2   NKDGVLISEG QGFMTREFLK SLRKPFGDFM EPKFEFAVKF NALELDDSDL
macmPPARG   NKDGVLISEG QGFMTREFLK SLRKPFGDFM EPKFEFAVKF NALELDDSDL
drPPARG     NKDGTLISYG QIFMTREFLK SLRKPFCEMM EPKFEFSIKF NMLELDDCDM
hsPPARGi1   NKDGVLISEG QGFMTREFLK SLRKPFGDFM EPKFEFAVKF NALELDDSDL
btPPARGi1   NKDGVLISEG QGFMTREFLK SLRKPFGDFM EPKFEFAVKF NALELDDSDL
xlPPARG     NKDGVLVAEG QGFMTREFLK SLRKPFSDFM EPKFEFAIRF NSLELDDSDL
olPPARG     NKDGTLISYG QIFMTREFLK SLRKPFCQML EPKFDFSVKF NTLELDDSDM
salsPPARG   NKDGTLFAYG QIFMTREFLK SLRKPFCEMM EPKFEFAAKF NLLELDDSDM
omPPARG     NKDGTLFAYG QIFMTREFLK SLRKPFCEMM EPKFEFAVKF NVLELDDSDM
Consistency ****89886* *6******** ******5879 ****9*999* *7*****9*8
```

```
            .........560.........570.........580.........590.........600
mmPPARGi1   AIFIAVIILS GDRPGLLNVK PIEDIQDNLL QALELQLKLN HPESSQLFAK
rnPPARGi2   AIFIAVIILS GDRPGLLNVK PIEDIQDNLL QALELQLKLN HPESSQLFAK
macfPPARG   AIFIAVIILS GDRPGLLNVK PIEDIQDNLL QALELQLKLN HPESSQLFAK
ggPPARG     AIFIAVIILS GDRPGLLNVK PIEDIQDNLL QALELQLKLN HPESSQLFAK
ocPPARG     AIFIAVIILS GDRPGLLNVK PIEDIQDNLL QALELQLKLN HPEASQLFAK
mmPARGi2    AIFIAVIILS GDRPGLLNVK PIEDIQDNLL QALELQLKLN HPESSQLFAK
rnPPARGi1   AIFIAVIILS GDRPGLLNVK PIEDIQDNLL QALELQLKLN HPESSQLFAK
btPPARGi2   AIFIAVIILS GDRPGLLNVK PIEDIQDNLL QALELQLKLN HPESSQLFAK
clPPARG     AIFIAVIILS GDRPGLLNVK PIEDIQDNLL QALELQLKLN HPESSQLFAK
ssPPARG     AIFIAVIILS GDRPGLLNVK PIEDIQDNLL QALELQLKLN HPESSQLFAK
hsPPARGi2   AIFIAVIILS GDRPGLLNVK PIEDIQDNLL QALELQLKLN HPESSQLFAK
macmPPARG   AIFIAVIILS GDRPGLLNVK PIEDIQDNLL QALELQLKLN HPESSQLFAK
drPPARG     ALFLAVIILS GDRPGLLDVK PIEDLQETVL HSLELQLKIN HPDSLQLFAK
hsPPARGi1   AIFIAVIILS GDRPGLLNVK PIEDIQDNLL QALELQLKLN HPESSQLFAK
btPPARGi1   AIFIAVIILS GDRPGLLNVK PIEDIQDNLL QALELQLKLN HPESSQLFAK
xlPPARG     AIFVAVIILS GDRPGLLNVK PIEDIQDSLL QALELQLKLN HPDSAQLFAK
olPPARG     ALFLAVIILS GDRPGLLNVK PIEQLQETVL HSLELHLKIN HPDSLQLFAK
salsPPARG   ALFFAVIILS GDRPGLVNVK PIEDLQETVL QALELQLKTI HPDCPQLFAK
omPPARG     ALFFAVIILS GDRPGLVNVK PIEDLQETVL QALELQLKTI HPDCPQLFAK
Consistency *9*8****** ******99** ***99*878* 89***9**88 **886*****
```

```
            .........610.........620.........630.........640...
mmPPARGi1   VLQKMTDLRQ IVTEHVQLLH VIKKTETDMS LHPLLQEIYK DLY
rnPPARGi2   VLQKMTDLRQ IVTEHVQLLH VIKKTETDMS LHPLLQEIYK DLY
macfPPARG   LLQKMTDLRQ IVTEHVQLLQ VIKKTETDMS LHPLLQEIYK DLY
ggPPARG     LLQKMTDLRQ IVTEHVQLLQ IIKKTETDMS LHPLLQEIYK DLY
ocPPARG     LLQKMTDLRQ IVTEHVQLLQ VIKKTETDMS LHPLLQEIYK DLY
mmPARGi2    VLQKMTDLRQ IVTEHVQLLH VIKKTETDMS LHPLLQEIYK DLY
rnPPARGi1   VLQKMTDLRQ IVTEHVQLLH VIKKTETDMS LHPLLQEIYK DLY
btPPARGi2   LLQKMTDLRQ IVTEHVQLLQ VIKKTETDMS LHPLLQEIYK DLY
clPPARG     LLQKMTDLRQ IVTEHVQLLQ VIKKTETDMS LHPLLQEIYK DLY
ssPPARG     LLQKMTDLRQ IVTEHVQLLQ VIKKTETDMS LHPLLQEIYK DLY
hsPPARGi2   LLQKMTDLRQ IVTEHVQLLQ VIKKTETDMS LHPLLQEIYK DLY
macmPPARG   LLQKMTDLRQ IVTEHVQLLQ VIKKTETDMS LHPLLQEIYK DLY
drPPARG     VLQKMTDLRQ LVTDHVQLIQ MMKETEADWS LHPLLQEIMR DLY
hsPPARGi1   LLQKMTDLRQ IVTEHVQLLQ VIKKTETDMS LHPLLQEIYK DLY
btPPARGi1   LLQKMTDLRQ IVTEHVQLLQ VIKKTETDMS LHPLLQEIYK DLY
xlPPARG     LLQKMTDLRQ VVTEHVQLLQ LIKKTEADMC LHPLLQEIYK DLY
olPPARG     VLQKMTDLRQ MVTDHVQLIQ LLKKTEVDMF LHPLLQEIMK DLY
salsPPARG   LLQKMTDLRQ LVANHVRHIH LLKKQELQMC LHPLLQEIMR DLY
omPPARG     LLQKMTDLRQ LVANHVRLIH LLKKQELEVC LHPLLQEIMR DLY
Consistency 8********* 8*88**9997 88*98*7987 ********79 ***
```

Supplemental data 3A: Synteny of zebrafish genomic regions of Pparaa and Pparab to human genomic region of PPARA

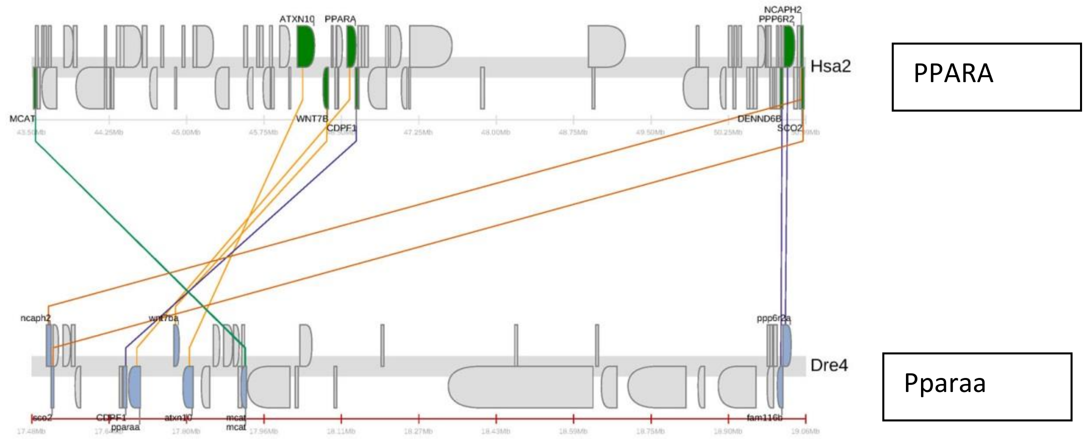

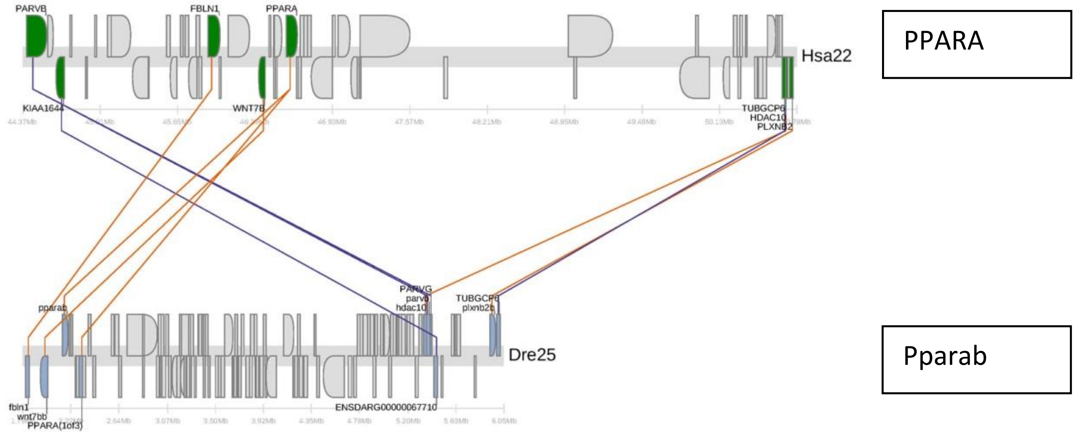

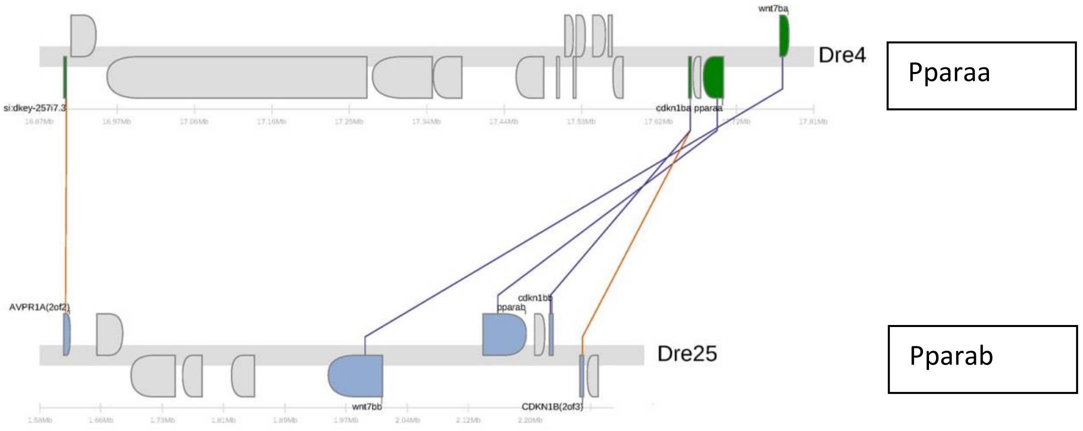

Supplemental data 3B: Synteny of zebrafish genomic regions of Pparda and Ppardb to human genomic region of PPARD

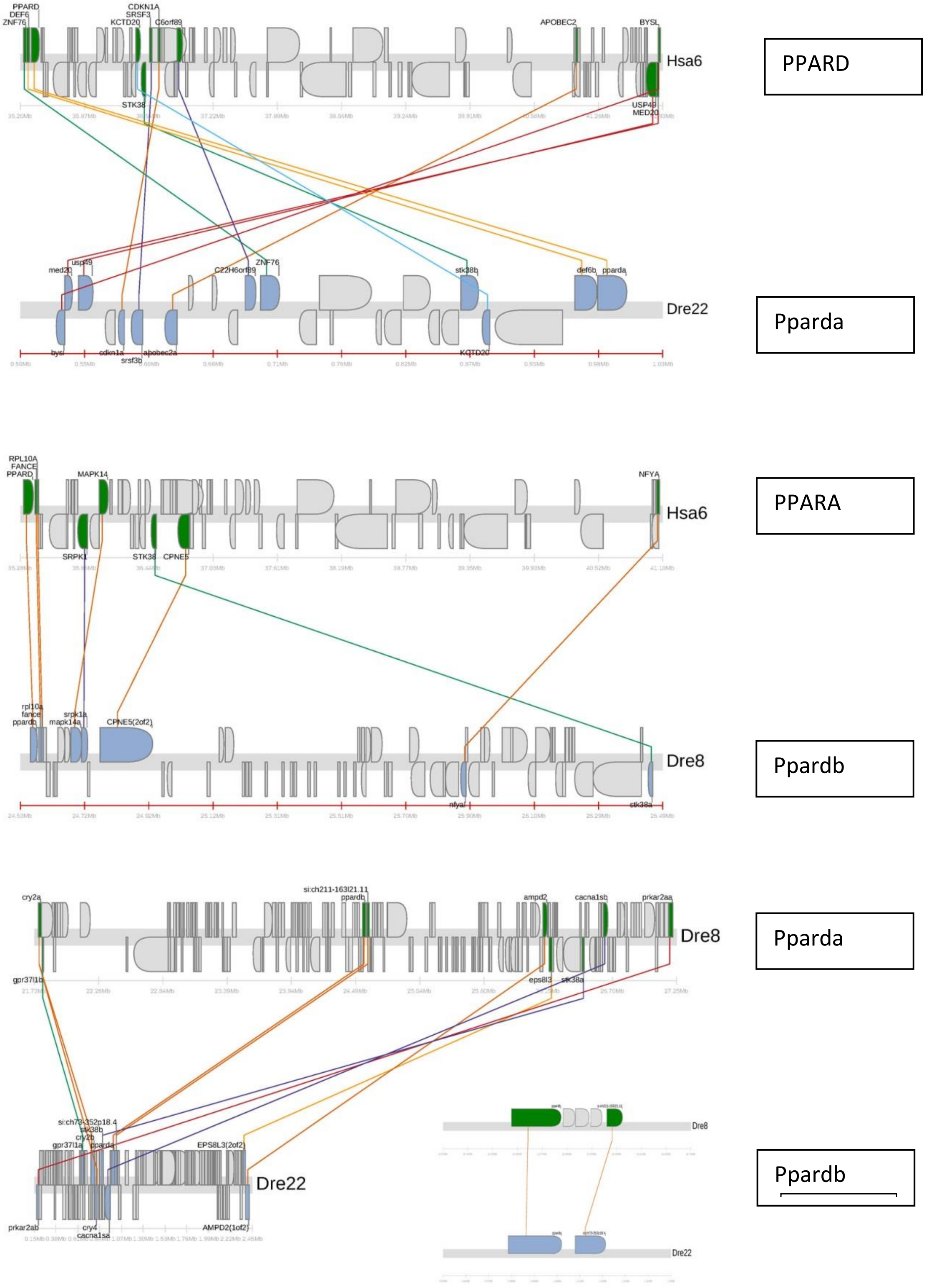

Supplemental data 3C: Synteny of zebrafish genomic regions of Pparg to human genomic region of PPARG

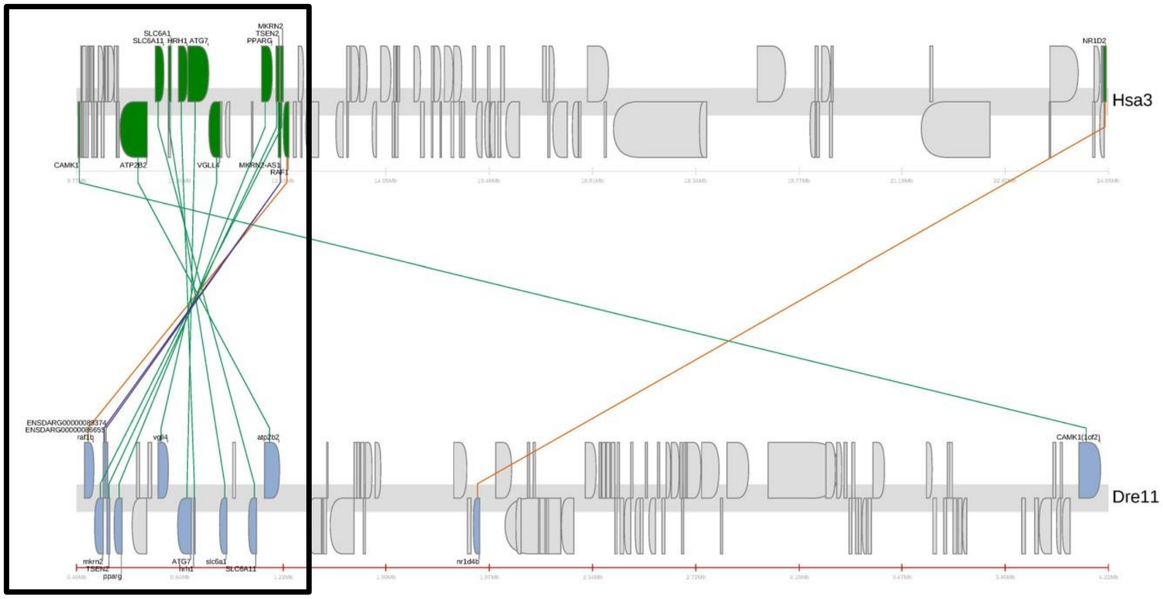

PPARG

Pparg

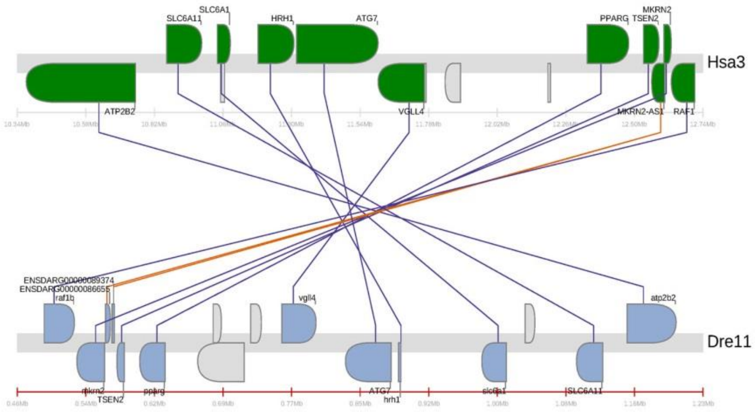

Zoom in at indicated region
